# Exercise-induced peptide TAG-23 protects cardiomyocytes from reperfusion injury through regulating PKG–cCbl interaction

**DOI:** 10.1007/s00395-021-00878-4

**Published:** 2021-06-25

**Authors:** Zijie Cheng, Hao Zhang, Li Zhang, Xuejun Wang, Qijun Zhang, Mengwen Feng, Deliang Hu, Hua Li, Lingmei Qian

**Affiliations:** 1 Department of Cardiology, The First Affiliated Hospital of Nanjing Medical University, NO.300 Guangzhou Road, Gulou District, Nanjing, 210029 China; 2 Department of Cardiology, Tongren Hospital Affiliated To Medical School of Shanghai Jiao Tong University, Shanghai, China; 3 Department of Cardiology, Zhongshan Hospital, Fudan University, Shanghai, China; 4 Department of Internal Medicine, The Affiliated Drum Tower Hospital of Nanjing University Medical School, Nanjing, China; 5 Department of Cardiology, The Affiliated People’s Hospital of Ningbo University, Ningbo, China; 6 Department of Emergency, The First Affiliated Hospital of Nanjing Medical University, Nanjing, China

**Keywords:** TAG-23, PKG, cCbl, Doxorubicin, Myocardial infarction

## Abstract

Recent studies have revealed that proper exercise can reduce the risk of chronic disease and is beneficial to the body. Peptides have been shown to play an important role in various pathological processes, including cardiovascular diseases. However, little is known about the role of exercise-induced peptides in cardiovascular disease. We aimed to explore the function and mechanism of TAG-23 peptide in reperfusion injury and oxidative stress. Treatment with TAG-23 peptide significantly improved cell viability, the mitochondrial membrane potential, and ROS levels and reduced LDH release, the apoptosis rate and caspase 3 activation in vitro. In vivo, TAG-23 ameliorated MI and heart failure induced by I/R or DOX treatment. Pull-down assays showed that TAG-23 can bind to PKG . The TAG-23-PKG complex inhibited PKG degradation through the UPS. We also identified cCbl as the E3 ligase of PKG and found that the interaction between these proteins was impaired by TAG-23 treatment. In addition, we provided evidence that TAG-23 mediated Lys48-linked polyubiquitination and subsequent proteasomal degradation. Our results reveal that a novel exercise-induced peptide, TAG-23, can inhibit PKG degradation by serving as a competitive binding peptide to attenuate the formation of the PKG–cCbl complex. Treatment with TAG-23 may be a new therapeutic approach for reperfusion injury.

**Supplementary Information:**

The online version contains supplementary material available at 10.1007/s00395-021-00878-4.

## Introduction

Myocardial infarction (MI) is the leading cause of death worldwide [8, 33]. Emerging evidence have demonstrated that apoptosis, the inflammatory response, and reactive oxygen species (ROS) are associated with cellular damage during the ischemic period [1]. Cardiomyocyte death resulting from reperfusion injury can be prevented at the end of the ischemic time, and targeting regulated cell death pathways before reperfusion injury appears to be a promising approach to minimize MI injury [4].

It is well established that exercise not only reduces the risks of chronic disease but also provides protection against cardiovascular disease [5]. Exercise is recommended for patients with heart failure or following MI [34]. Recent studies have shown that the mechanism underlying exercise-induced cardioprotection mainly contributes to increasing antioxidant capacity, anti-inflammatory effects, and the secretion of beneficial hormones or cytokines [16, 23]. Exercise-induced bioactive factors, including peptides, hormones, and nucleic acids, are secreted into the circulatory system and allow cell communication within different organs [12, 15]. These bioactive components may be beneficial for physiological function. However, the underlying mechanisms of exercise-induced cardioprotection remain unknown.

Accumulating evidence has shown that peptides play important roles in various pathophysiological processes. For example, Bβ_15–42_ can ameliorate leukocyte transmigration across the endothelial monolayer to ameliorate ischemia–reperfusion (I/R) injury [24]. Oral administration of the peptide Val-Pro-Pro, which is derived from casein, reduces proinflammatory macrophage accumulation in adipose tissue [1]. Pretreatment with GLP-1 peptide protects against ischemic cardiac dysfunction and improves heart function during reperfusion [21]. These studies emphasize the importance of peptides in pathological processes. Despite advances in the knowledge of cardiovascular diseases, further identification of bioactive peptides will facilitate better understanding of the mechanism of I/R injury.

Previous studies have quantified peptides that are differentially induced by exercise by 2D-LC–MS/MS, and 5548 unique peptides that are induced at different time points have been screened [23]. In the peptidome, the endogenous peptide TAG-23 attracted our attention because it is upregulated in the plasma during exercise. Live-cell microscopy was utilized to observe the effect of TAG-23 on migration. A wound healing assay showed that compared with a scramble peptide, TAG-23 peptide can increase cell migration by affecting the cell cycle and extracellular matrix remodeling [23]. TAG-23 peptide is derived from transgelin, which is primarily expressed in smooth muscle cells. Transgelin knockout mice show decreased actin content [39]. Transgelin, which is an androgen receptor (AR) inhibitor, can suppress AR function in LNCaP cells by binding with p53 [41]. To date, there have been no reports regarding the role of TAG-23 peptide in cardiovascular disease.

In this study, we aimed to evaluate the function and mechanism of the exercise-induced peptide TAG-23 in reperfusion and oxidative stress. Our study demonstrated that TAG-23 confers cardioprotective effects against MI and serves as a competitive peptide to attenuate the PKG–cCbl interaction. We also provide evidence that TAG-23 mediates PKG degradation at the Lys48 site. Taken together, these results are the first to demonstrate the function and mechanism of exercise-induced peptides in cardiovascular disease and provide a new clue for understanding MI.

## Materials and methods

### Cell culture

Rat primary cardiomyocytes were extracted one day after birth. The ventricles were harvested, rinsed briefly in 75% ethanol and maintained in DMEM. After the heart tissues were cut into small fragments, they were washed twice with PBS and then transferred to digestion solution (0.4% type 2 collagenase/0.6% pancreatin). Repeat digestion was performed at 37 °C for 20 min. The supernatant was collected after every round of digestion, and the cells were centrifuged at 1000 rpm for 5 min. Cardiac fibroblasts were minimized by pre-plating the cells for 2 h. Cardiomyocytes in the suspension were seeded on gelatin-coated culture plates (G1393, Sigma-Aldrich, USA). The cells were then cultured on gelatinized plates for 24–48 h before further study.

To induce hypoxia/reperfusion (H/R), injury cells were treated with H/R buffer in a hypoxic chamber (Billups-Rothenberg), and the chamber was flushed with 95% N_2/_5% CO_2_ for 30 min. Then, the chamber was closed for an additional 4 h. The cells were washed twice with PBS, and the culture medium was replenished. Neonatal rat ventricular myocytes (NRVMs) incubated in normoxic medium (in mM: 4 HEPES, 137 NaCl, 3.8 KCl, 0.9 CaCl_2_, 0.49 MgCl_2_, and 5.6 D-glucose, pH 7.4) were used as controls.

The sequence of TAG-23 was MGSNRGASQAGMTGYGRPRQIIS (TAG-23). The scramble peptide was RGMINGRMIQSTGSYPSARGQAG (control). The peptides were synthesized by Scientific Peptide Biological Technology Co., Ltd. (Shanghai, China). The concentration used is indicated in the figure legends. The peptides were added to the culture medium for 1 h before H/R treatment.

### JC-1 assay

The mitochondrial membrane potential was measured using a mitochondrial membrane potential assay kit with JC-1 according to the manufacturer’s instructions. The NRVMs were cultured in serum-free DMEM containing (1 ×) JC-1 staining working solution at 37 °C for 20 min. Then, the cells were washed twice with JC-1 buffer, 2 ml of DMEM was added, and the cells were photographed with a fluorescence microscope (BX61; Olympus Corporation, Tokyo, Japan).

### ROS levels

The levels of intracellular ROS were determined using a ROS assay kit (Beyotime, Shanghai, China) according to the instructions. NRVMs were incubated in serum-free DMEM containing 0.1% 2’,7’-dichlorofluorescein diacetate (DCFH-DA) at 37 °C for 20 min, washed three times with serum-free DMEM and photographed with a fluorescence microscope.

### TUNEL staining

NRVMs were seeded in 6-well plates (1 × 10^5^ cells per well). After H/R treatment, the cells were washed twice with PBS and fixed with 4% paraformaldehyde. Apoptotic cells were visualized by TUNEL staining according to the manufacturer’s protocol (Promega). The TUNEL fluorescence intensity/DAPI fluorescence intensity was used to calculate the percentage of positively stained cells, and the density was evaluated using Image J software 1.26 (Wayne Rasband, National Institutes of Health, Bethesda, MD, USA).

### Cell viability

NRVMs were seeded on 96-well plates (1500 cells/well). After 24 h, the cells were treated with different concentrations of TAG-23, and cell viability was measured by the CCK8 assay using a cell viability assay kit (Promega) according to the manufacturer’s protocols.

### MI

I/R injury was induced in wild-type male mice (8 weeks) on a C57/B6 background. Mice were sedated with 5% isoflurane‑O_2_ (cat.no. 792632; Sigma‑Aldrich; Merck KGaA) balanced mixture (maintained at 1.5%). All mice were anaesthetized by breathing a 5% isoflurane‑O_2_ balanced mixture (maintained at 1.5%). Mice were confirmed to be deeply anesthetized after they were immobile for 1 min. Left anterior descending occlusion was performed for 45 min. Then, 10 mg/Kg peptides were injected through the tail vein. The ligature was then removed, and the animals were maintained for 1 week of reperfusion. The mice were killed by inhalation of 25% CO_2_, until respiratory and cardiac arrest occurred. Then, 2,3,5-triphenyltetrazolium chloride (TTC) and Evans blue staining or tissue harvesting for histological staining were performed to analyze the infarct size and fibrosis rates. Plasma was collected to measure cTnI and creatine kinase (CK)-MB levels. All animal studies were approved by the Animal Care and Ethics Committee of Nanjing Medical University. The animal experiments were performed according to the guide for the Care and Use of Laboratory Animals.

### TTC + Evans blue

After 1 week of reperfusion, Evans blue and TTC dyes were used to determine the infarct size (IS) and area at risk (AAR). The mouse was anesthetized, and LAD was re-ligated. Then 2 ml 2% Evans blue was injected through right jugular vein. The heart was immediately removed and frozen and then sectioned into 1 mm-thick slices. The slices were incubated in 1.0% TTC for 15 min at 37 ℃. Serial sections of the heart were photographed, and infarct size was determined by computerized planimetry using ImageJ software. The infarct size was then calculated and quantified.

### Histological analysis

Hearts were harvested and immediately fixed in 4% paraformaldehyde for 48 h. The samples were dehydrated, embedded in paraffin and sectioned into 5-μm-thick slices on a sliding microtome (Leica, Nussloch, Germany). Fibrosis was detected by staining paraffin-embedded sections with Masson’s trichrome. Blue collagen staining was quantified using ImageJ software (version 1.52t, National Institutes of Health, Bethesda, MD, USA). The surface area of a single cardiomyocyte was measured using the Image J Program (v 1.52a, NIH, USA). The cardiomyocyte area was quantified by measuring 200 cells (randomly selected in 3 sections) in each heart with source of sample blinded. Only cells lying fully within the visual field were quantified.

### CK-MB, cTnI, and LDH measurement

Mouse cTnI plasma levels were measured using an ELISA kit from Life Diagnostics, Inc. (West Chester, PA). CK-MB concentrations were measured using an ELISA kit (LSBio, Seattle, WA, USA) according to the manufacturer’s protocol. The LDH concentration was also measured using an ELISA kit (Promega, Madison, WI, USA) according to the instructions. Cell supernatant and mouse serum were collected to analyze the LDH release.

### DOX-induced heart failure

Male C57BL/6J mice (6–10 weeks of age, 20 ~ 22 g) were obtained from the Model Animal Research Center of Nanjing University (Nanjing, Jiangsu, China), and all procedures were approved by the ethical committee of Nanjing Medical University. All animals were housed at 20 ~ 25 °C and 50 ~ 70% relative humidity. To induce heart failure, 5 mg/kg DOX was injected for 4 consecutive weeks (*i.p.*, on days 7, 14, 21 and 28). 10 mg/kg peptides were injected before DOX injection every time. Body weight was recorded every week. The mice were subjected to echocardiographic analysis 1 week after the last DOX injection and killed for further analysis. Mice were euthanatized by inhalation of 25% CO_2_, until respiratory and cardiac arrest occurred and killed for further analysis. All animal studies were approved by the Animal Care and Ethics Committee of Nanjing Medical University.

### Echocardiography

To evaluate cardiac function, mice were anesthetized with 1% isoflurane and analyzed with a Vevo 2100 High Resolution Imaging System. Cardiac contractile function in conscious, gently restrained mice was examined by echocardiography using a Vevo 2100 system (MS400C probe, Visual Sonics). Furthermore, ventricular fractional shortening (FS) was calculated as left ventricular internal diameter in diastole (LVIDd)—left ventricular internal diameter in systole (LVIDs)/LVIDd.

### Western blot analysis

Proteins were isolated from cells using lysis buffer (RIPA and protease inhibitor cocktail). Protein quantification was performed using a BCA protein detection kit (23,229; Thermo Fisher Scientific). The same volume of protein from each sample was separated on 10% SDS-PAGE gels and transferred to nitrocellulose membranes (Millipore, Billerica, MA, USA). The membranes were blocked with 5% milk and then incubated with primary antibodies overnight. Detailed antibody information is listed in Supple Table S3. A FluorChem M system (ProteinSimple, San Jose, CA, USA) was used to quantify bands corresponding to proteins involved in the orchestrated immune response.

### Real-time PCR

Total RNA was extracted from cells using TRIzol reagent (Thermo Fisher Scientific). The concentration of RNA was determined by measuring the absorbance ratio of 260/280 nm using a NanoDrop ND-1000 spectrophotometer (Thermo Fisher Scientific). RNA was reverse transcribed using the PrimeScript™ RT Reagent Kit with gDNA Eraser (RR047A; Takara, Tokyo, Japan), and cDNA was analyzed by qRT-PCR using SYBR^®^ Premix Ex Taq™ (RR420A; Takara, Tokyo, Japan). The data were normalized to the levels of GAPDH and further analyzed using the 2^−ΔΔCT^ method. The sequences for all the primers used for qPCR are listed in the Supple Table S4.

### Biotin pull-down assay

Biotinylated scramble and biotinylated TAG-23 peptides were synthesized by Scientific Peptide Biological Technology Co., Ltd. (Shanghai, China). The biotinylated scramble peptide was used as a control, and a total of 2 µg of biotinylated peptide was added to the lysate containing Dynabeads™ M-280 Streptavidin Beads (Invitrogen) overnight at 4 ℃. Whole cell lysates were cleared by centrifugation at 12,000 rpm for 30 min at 4 °C and then precleared with Dynabeads™ M-280 Streptavidin Beads to eliminate nonspecific binding. Then, the precleared proteins were incubated with bead-biotinylated peptide complexes overnight at 4 °C. The beads were washed extensively with wash buffer (0.1% Triton X-100, 50 mM Tris–HCl, 300 mM NaCl, 5 mM EDTA, and 0.02% sodium azide, pH 7.4), and the beads/column were washed with 4 × 1 mL of ice-cold buffer followed by 2 × 1 ml ice-cold PBS. Glycine (0.1 M, pH ~ 2.5) was used to elute the peptide complexes. The complexes were resolved by 10% SDS-PAGE for subsequent silver staining.

### Silver staining and mass spectrometry

After 10% SDS-PAGE, the gel was maintained in a clean plastic 15-cm dish. Silver staining was performed by strictly following the manufacturer’s protocol (Pierce^®^ Silver Stain for Mass Spectrometry, 24,600, USA). When the band was visible, the developer working solution was immediately replaced with stop solution, and the gel bands were excised for further mass spectrometry analysis (Shanghai Applied Protein Technology Co., Ltd) on a Q Exactive mass spectrometer (Proxeon Biosystems, now Thermo Fisher Scientific).

### RNA-seq analysis

Total RNA was extracted using TRIzol reagent (Life Technologies, Carlsbad, CA, US) following the manufacturer’s instructions, and the RNA integrity number (RIN) was evaluated using an Agilent Bioanalyzer 2100 (Agilent Technologies, Santa Clara, CA, US) to determine RNA integrity. Qualified total RNA was further purified using a RNeasy mini kit (QIAGEN, GmBH, Germany) and an RNase-Free DNase Set (QIAGEN, GmBH, Germany). Total RNA was amplified and labeled with the Low Input Quick Amp Labeling Kit, One-Color (Agilent Technologies, Santa Clara, CA, US). Data were extracted with TapeStation Analysis Software A.02.01 SR1.

### Immunoprecipitation

For ubiquitination assay, different plasmids were transfected to H293 or H9C2 cells. After 48 h transfection, 10 µm MG132 was pretreatment for 2 h and then cells were collected for further analysis. Cells were lysed at 4 °C using lysis buffer (50 mM Tris–HCl, 150 mM NaCl, 1 mM EDTA, 10 mM NaF, 1 mM sodium vanadate, and 0.5% NP-40, pH 7.5) containing protease inhibitor cocktail (Roche). Whole cell lysates were cleared by centrifugation at 12,000 rpm for 30 min at 4 °C and then precleared with protein A/G-Magnetic Beads (Thermo Scientific Fisher) to eliminate nonspecific binding. The indicated antibodies and Protein A/G-Magnetic Beads (Thermo Scientific Fisher) were incubated overnight at 4 °C. Then, the precleared proteins were incubated with bead-antibody complexes overnight at 4 °C. The beads were washed extensively with wash buffer (0.1% Triton X-100, 50 mM Tris–HCl, 300 mM NaCl, 5 mM EDTA, and 0.02% sodium azide, pH 7.4), and the beads/column were washed 4 × 1 mL of ice-cold buffer followed by 2 × 1 ml ice-cold PBS. The proteins were resolved by 8–12% SDS-PAGE for subsequent western blotting.

### Statistical analysis

The data are expressed as the mean ± SD unless otherwise indicated. Statistical differences were analyzed by unpaired 2-sided Student’s *t* test or 2-way ANOVA with Bonferroni correction for multiple comparisons. Data analysis was performed with GraphPad Prism software version 8.0. A value of *P* ≤ 0.05 was considered statistically significant.

## Results

### Function of TAG-23 in cardiomyocytes exposed to H/R

**Fig. 1:**
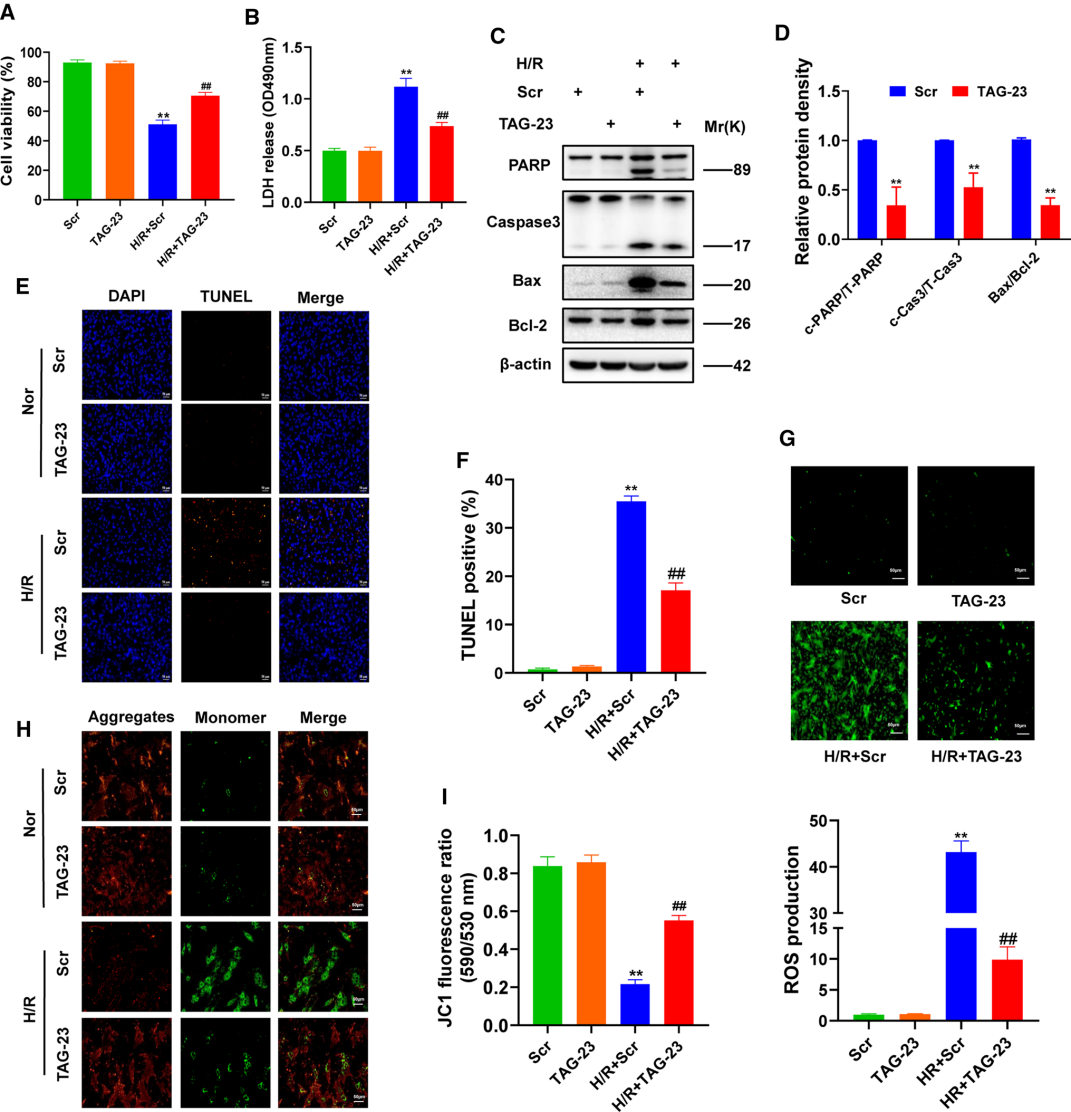
Function of TAG-23 in cardiomyocytes exposed to I/R. **A** The cell viability was measured in H/R model. TAG-23 increased the cell viability compared with scramble peptide group. *n* = 3 per group (two-way ANOVA analysis with Bonferroni’s multiple comparison test). **B** LDH release was measured in H/R model. TAG-23 reduced the LDH release compared with scramble peptide group. *n* = 3 per group (two-way ANOVA analysis with Bonferroni’s multiple comparison test). **C** Apoptosis markers (PARP, Caspase3, Bax, Bcl-2) were measured by western blot. TAG-23 significantly inhibits H/R-induced cell apoptosis. **D** Quantification data of western blot in H/R group. *n* = 3 per group (Student’s *t* test). **E** Representative photography of TUNEL assay. **F** Quantification data of TUNEL assay. TUNEL results revealed that TAG-23 inhibited the cell apoptosis induced by H/R exposure. *n* = 3 per group (two-way ANOVA analysis with Bonferroni’s multiple comparison test). **G** Representative photography of ROS contents (up) and quantification data (down). *n* = 3 per group (two-way ANOVA analysis with Bonferroni’s multiple comparison test). **H** Representative photography of mitochondrial membrane potential in H/R model. **I** JC-1 was measured under 590/530 nm. *n* = 3 per group (two-way ANOVA analysis with Bonferroni’s multiple comparison test). **P < 0.01, ***P < 0.001. Data are means ± SD with *n* = 3 independent biological cultures

To study the function of TAG-23 peptide, we first constructed an H/R model which is a well-established model for studying ischemia reperfusion injury [35]. Z-VAD, an apoptosis inhibitor, served as a positive control [14]. Cell viability was significantly reduced after H/R treatment, and Z-VAD restored apoptosis (Supple Fig. 1A). To explore the localization of TAG-23 peptide, we synthesized FITC-labeled TAG-23 and added it to the culture medium for 2 h. The results showed that TAG-23 peptide was mainly located in the cytoplasm (Supple Fig. 1B). We then evaluated the cardiomyocytes toxicity through CCK8 assay. Different concentrations of TAG-23 peptide had no influence under normoxic conditions (Supple Fig. 1C). In this study, we synthesized a scramble peptide to serve as a control peptide. There was no significant difference in cell viability upon treatment with different concentrations of the scramble peptide (Supple Fig. 1D). TAG-23 blocked H/R-induced cell damage, as evidenced by cell viability and LDH release (Fig. 1A, B). In addition, we assessed whether different concentrations of TAG-23 can influence cell viability and LDH release. TAG-23 intervention had a dose-dependent effect (Supple Fig. 1E, F). Thus, our study is the first to report that TAG-23 may protect against hypoxia reperfusion injury.

### TAG-23 attenuates apoptosis and protects mitochondrial function

To further study the function of TAG-23 in H/R injury, apoptosis was evaluated, and reductions in cleaved-caspase3 activation, cleaved-PARP activation (Fig. 1C, D). TUNEL staining results showed that treatment of TAG-23 significantly reduced cell apoptosis (Fig. 1E, F). Notably, compared with the scramble peptide, the TAG-23 peptide reduced ROS content in the H/R model (Fig. 1G). TAG-23 significantly reduced the mitochondrial membrane potential, as evidenced by the ratio of JC-1 monomers to aggregates (Fig. 1H), which is a marker of early apoptosis. We also provided evidence that TAG-23 protects the mitochondrial potential by measuring JC-1 fluorescence (Fig. 1I). Thus, our results revealed that TAG-23 can attenuate cell apoptosis and protect mitochondrial function.

### TAG-23 ameliorates MI injury

**Fig. 2:**
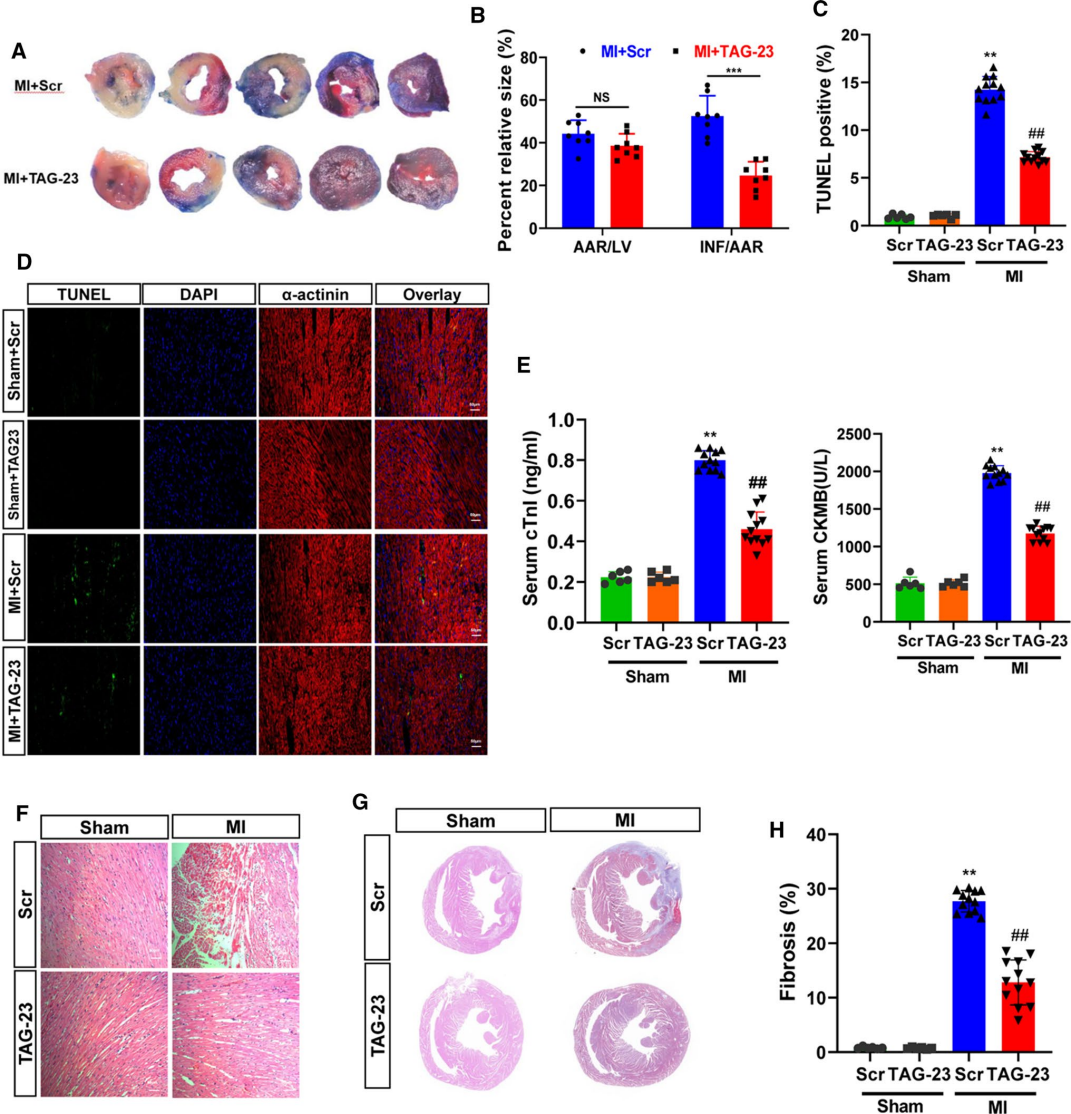
TAG-23 ameliorates the myocardial infarction injury. **A** Representative photographs of infarct size via TTC and Evans blue. **B** Quantification data of infarct size. *n* = 8 per group (Student’s *t* test). 10 mg/Kg TAG-23 significantly reduced the infarction area compared with scramble peptide group. **C** Quantitative data for TUNEL staining. *n* = 6 per group in sham group and *n* = 12 in MI group (two-way ANOVA analysis with Bonferroni’s multiple comparison test). **D** Representative photographs of TUNEL staining. TUNEL results demonstrated that TAG-23 inhibited cell apoptosis induced by myocardial infarction reperfusion injury. **E** Serum CK-MB and cTnI concentrations measured by ELISA. *n* = 6 per group in sham group and *n* = 12 in MI group (two-way ANOVA analysis with Bonferroni’s multiple comparison test). **F** Representative photographs of HE staining of heart sections. HE results showed that TAG-23 maintained the shape and size of myocardium fibers. **G** Representative photographs of Masson trichrome staining of heart sections. **H** Quantitative data for Masson staining. *n* = 6 per group in sham group and *n* = 12 in MI group (two-way ANOVA analysis with Bonferroni’s multiple comparison test). **P < 0.01, ***P < 0.001. Data are means ± SD with *n* = 6/12 independent biological replicates

To study the function of TAG-23 in vivo, we established a MI model. After a 45-min ischemia period, the TAG-23 peptide was injected through the tail vein. The, the animals underwent reperfusion for 1 week. The hearts were harvested for further study (Supple Fig. 2A). The infarct size was significantly decreased in the TAG-23 peptide intervention group by TTC and Evans blues assay (Fig. 2A, B). Notably, the TAG-23 group shows apoptosis reduction, as indicated by a reduction in TUNEL staining (Fig. 2C, D). MI-induced injury was profoundly ameliorated in the TAG-23 intervention group, as evidenced by the decrease in serum cTnI and CK-MB levels (Fig. 2E). The histological images of MI tissue showed disorganized myocardial fibers and disarrayed cardiomyocytes; however, TAG-23 intervention maintained the shape of myocardial fibers and less neutrophil infiltration (Fig. 2F). The fibrosis rate, which was detected by Masson staining, revealed that cardiac fibrosis was significantly suppressed by TAG-23 (Fig. 2G, H). These results revealed that TAG-23 plays a cardioprotective role in vivo in MI.

### TAG-23 attenuates DOX-induced heart failure

**Fig. 3:**
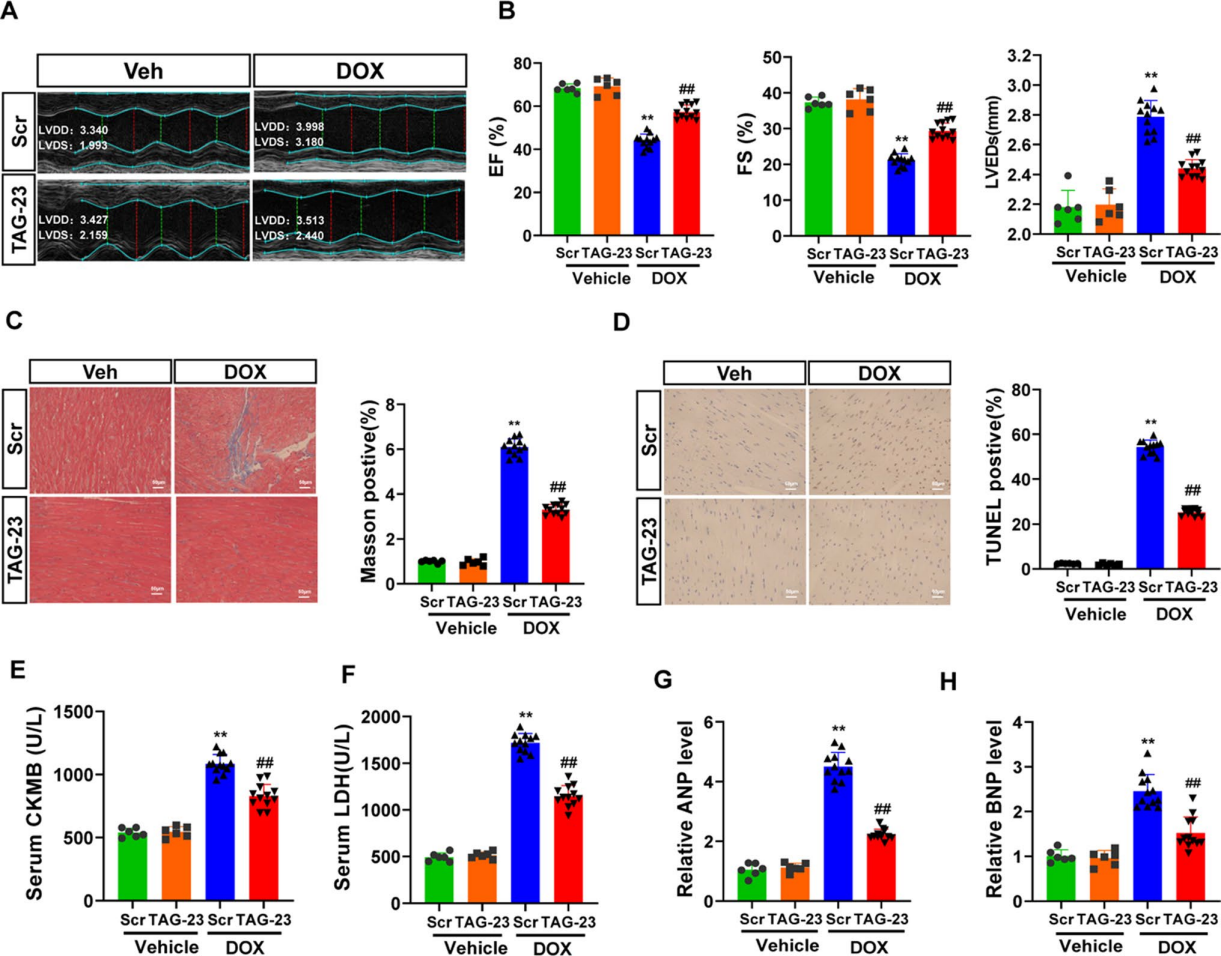
TAG-23 attenuates the DOX-induced heart failure. **A** Representative photographs of echocardiography of DOX-induced cardiac damage. TAG-23 maintained the cardiac function after DOX injection. **B** Quantitative data for echocardiography (*n* = 6 mice per group in vehicle group and *n* = 12 in DOX group, two-way ANOVA analysis with Bonferroni’s multiple comparison test, EF, FS, LVEDs). **C** Representative photographs of Masson trichrome staining of heart sections (left) and quantification data (right). *n* = 6 mice per group in vehicle group and *n* = 12 in DOX group (two-way ANOVA analysis with Bonferroni’s multiple comparison test). **D** Representative photographs of TUNEL staining (left) and quantification data (right). *n* = 6 mice per group in vehicle group and *n* = 12 in DOX group (two-way ANOVA analysis with Bonferroni’s multiple comparison test). **E** Serum CK-MB concentrations measured by ELISA. *n* = 6 mice per group in vehicle group and *n* = 12 in DOX group (two-way ANOVA analysis with Bonferroni’s multiple comparison test). **F** Serum LDH concentrations measured by LDH kit. *n* = 6 mice per group in vehicle group and *n* = 12 in DOX group (two-way ANOVA analysis with Bonferroni’s multiple comparison test) G-H ANP and BNP mRNA level was measured via qPCR. *n* = 6 mice per group in vehicle group and *n* = 12 in DOX group (two-way ANOVA analysis with Bonferroni’s multiple comparison test). **P < 0.01, ***P < 0.001. Data are means ± SD with *n* = 6/12 independent biological replicates

Previous studies have reported that DOX, a chemotherapeutic agent, can also trigger excessive loss of cardiomyocytes and lead to heart failure. Increasing evidence has demonstrated that oxidative stress is, at least in part, responsible for the DOX-induced cardiac injury. TAG-23 was injected through the tail vein every time before DOX treatment [40]. DOX (5 mg/kg) was intraperitoneally injected for 4 consecutive weeks to induce cardiomyocyte loss (Supple Fig. 2B). DOX-induced cardiac function was remarkably decreased in the scramble + DOX group; however, TAG-23 intervention significantly attenuated the echocardiographic phenotype (Fig. 3A). DOX-induced cardiac contractile dysfunction, which was indicated by decreases in ejection fraction (EF) and FS, and ventricular systolic dysfunction, which was indicated by the left ventricular end-systolic dimension (LVESd), were effectively improved in the TAG-23 injection group (Fig. 3B). However, there was no significant difference in left ventricular end-diastolic dimension (LVEDd) or left ventricular posterior wall thickness (LVPWd) (Supple Fig. 2C, Supple Table S1). Body weight was significantly decreased in the DOX injection group, whereas TAG-23 abolished this effect during DOX injection (Supple Fig. 2D). Notably, TAG-23 injection protected the heart from DOX-induced alterations in cardiac morphology and injury, as evidenced by cardiac fibrosis and the cardiomyocyte area (Fig. 3C, Supple Fig. 2E). The TUNEL assay showed that the apoptosis rates were decreased in the TAG-23 group, suggesting that TAG-23 exerted an antiapoptotic effect (Fig. 3D). DOX-induced cardiomyocyte damage was profoundly ameliorated in the TAG-23 intervention group, as indicated by decreased concentrations of serum CK-MB and LDH levels (Fig. 3E, F). Finally, we measured the mRNA levels of the cardiac injury markers atrial natriuretic peptide (ANP) and B-type natriuretic peptide (BNP), which were significantly attenuated in the TAG-23 injection group compared with the scramble peptide injection group (Fig. 3G, H). These data demonstrated that, similar to its role in MI, TAG-23 also plays a protective role in DOX-induced cardiac damage and heart failure.

### TAG-23 binds to PKG to activate the ERK signaling pathway

**Fig. 4:**
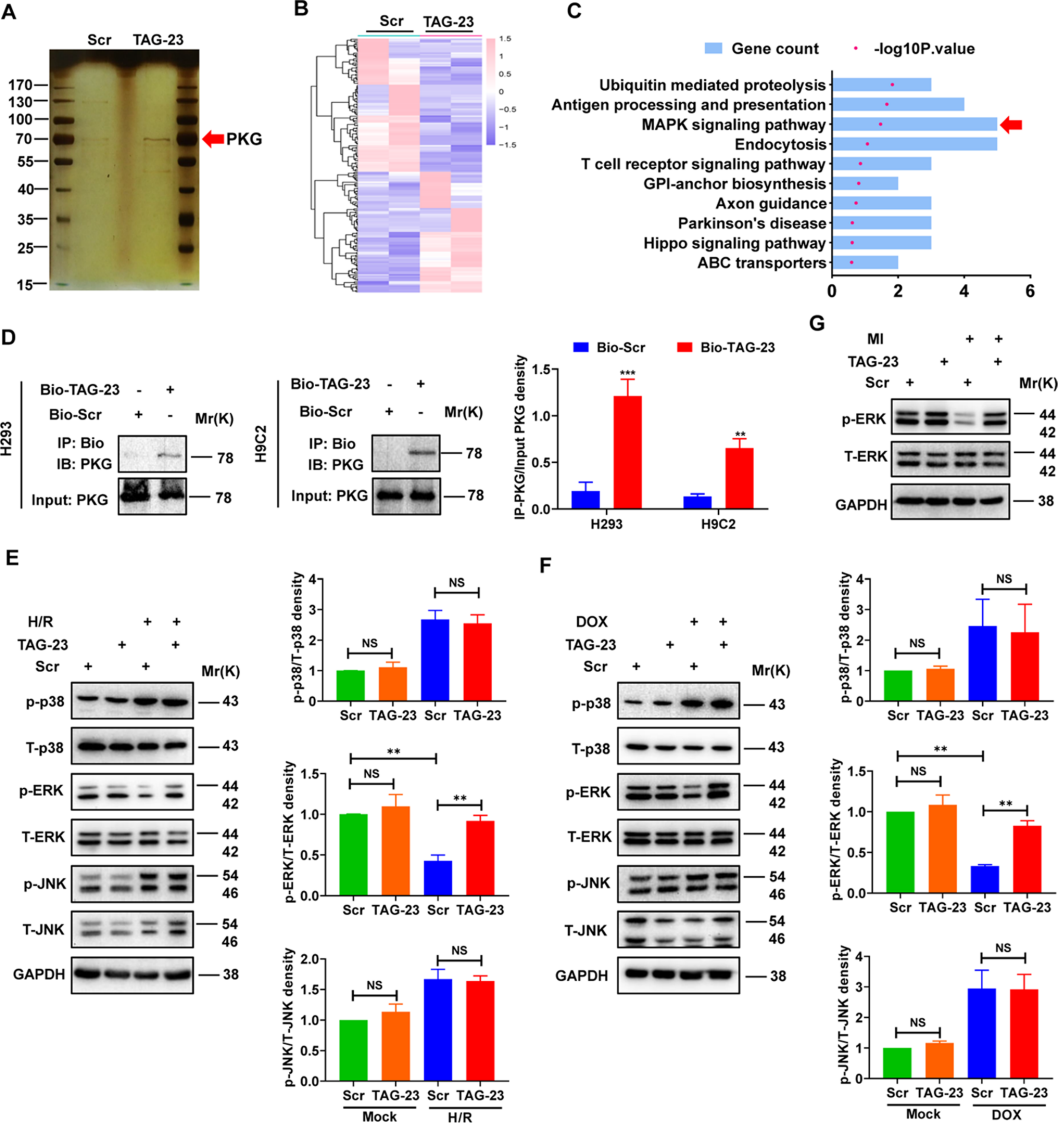
TAG-23 binds to PKG to activate the ERK signaling pathway. **A** Representative photographs of silver staining. **B** Heat map of RNA-seq. *n* = 2 per group, **c** KEGG analysis revealed that the top 10 pathways involved. **D** Interaction between TAG-23 and PKG in H293 cells and H9C2 cells. Co-IP assay was performed to verify the interaction between TAG-23 and PKG. *n* = 3 per group (Student’s *t* test). **E** MAPK signaling pathway was verified via western blot in H/R-induced hypoxia model. *n* = 3 per group (two-way ANOVA analysis with Bonferroni’s multiple comparison test). **F** MAPK signaling pathway was verified via western blot in DOX-induced oxidative stress. *n* = 3 per group (two-way ANOVA analysis with Bonferroni’s multiple comparison test). **G** ERK signaling pathway was verified via western blot in myocardial infarction tissues. TAG-23 significantly increased the ERK signaling pathway compared with scramble peptide group. *n* = 3 per group (two-way ANOVA analysis with Bonferroni’s multiple comparison test). **P < 0.01, ***P < 0.001. Data are means ± SD with *n* = 3 independent biological cultures

To investigate the mechanism of TAG-23 peptide involvement, we first performed a biotin pull-down assay. Due to the affinity of the peptide itself, we acquired a significant band that was approximately 70 ~ 80 kDa (Fig. 4A). Mass spectrometry analysis revealed the possible targets of biotinylated TAG-23, and the top 30 enriched proteins are listed in Supple Table S2. Among those enriched proteins, PKG, which has already been shown to protect against I/R injury, attracted our attention [13, 28]. We also analyzed the potential pathways by RNA-seq (Fig. 4B). Interestingly, KEGG analysis showed that TAG-23 peptide may be associated with Ub-mediated proteolysis and the MAPK signaling pathway (Fig. 4C). Previous studies have shown that PKG can regulate the MAPK signaling pathway [5, 27]. Thus, we speculated that there may be a relationship between TAG-23, PKG and MAPK. First, we incubated biotinylated peptides with proteins overnight and then performed a pull-down assay with streptavidin beads. The pulled-down proteins were then subjected to western blotting. We found that PKG was pulled down by biotinylated TAG-23 peptide in HEK cells and H9C2 cells (Fig. 4D). Next, we determined the downstream pathways of PKG and whether TAG-23 treatment can influence those pathways. Previous studies have shown that p38, ERK and JNK are downstream proteins of PKG [37]. NRVMs were treated with H/R and DOX, respectively. TAG-23 had no effect on p-p38 or p-JNK, and the total protein levels of p38 and JNK was unchanged (Fig. 4E, F, lane 3 vs. lane 4). However, there was a significant increase in the p-ERK level after TAG-23 intervention, whereas the level of p-ERK was notably downregulated in the scramble peptide group (Fig. 4E, F lane 3 vs. lane 4). We also verified the ERK signaling pathway in myocardial infarction tissues. TAG-23 can activate the p-ERK to protect against myocardial reperfusion injury (Fig. 4G).

### TAG-23 protects cardiomyocytes from reperfusion injury through ERK signaling pathway

**Fig. 5:**
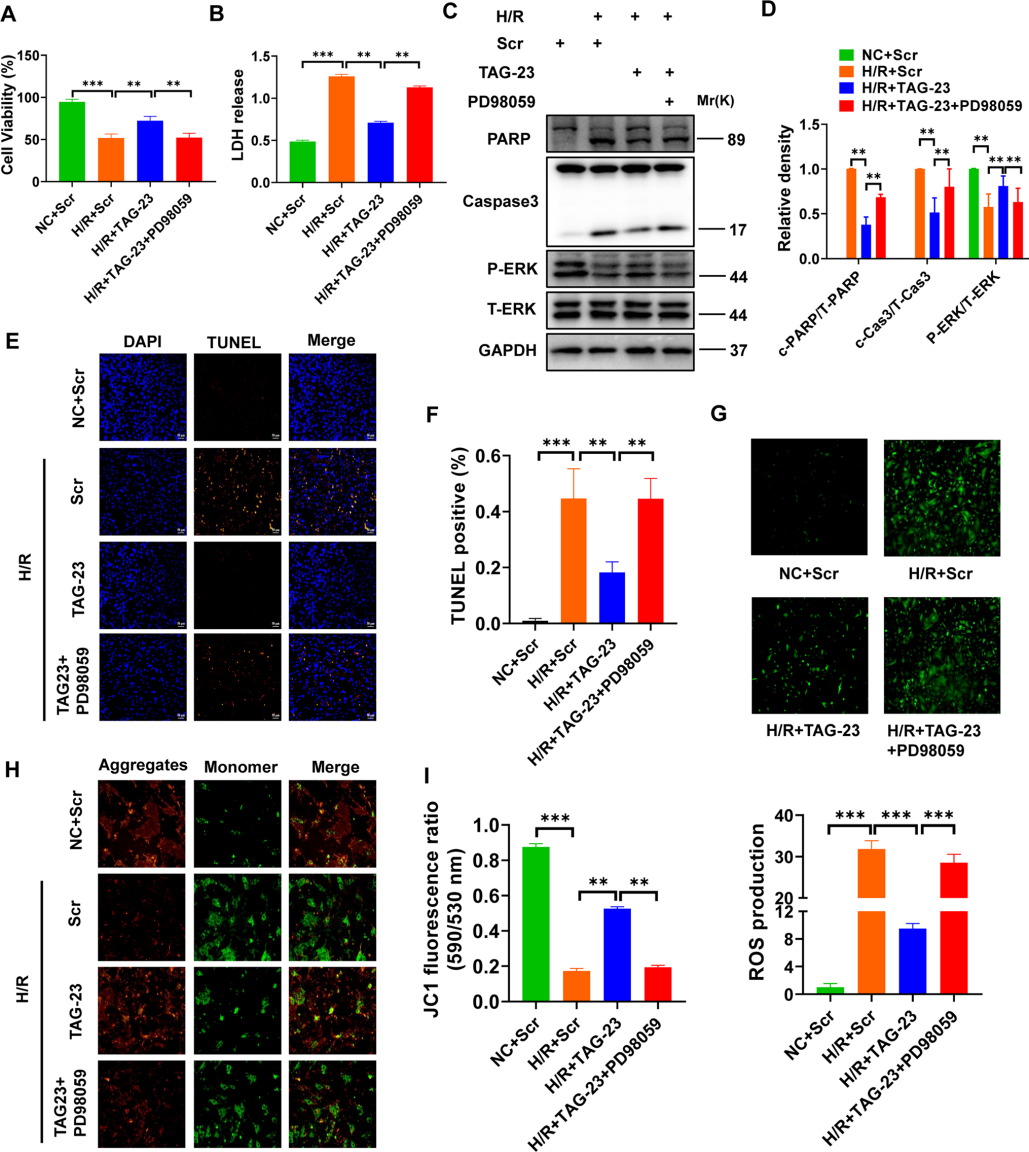
TAG-23 protects cardiomyocytes from reperfusion injury through ERK signaling pathway. **A** ERK inhibitor rescued the cell viability phenotype of TAG-23. *n* = 3 per group (one-way ANOVA analysis with Bonferroni’s multiple comparison test). **B** ERK inhibitor rescued the LDH release of TAG-23. *n* = 3 per group (one-way ANOVA analysis with Bonferroni’s multiple comparison test). **C** 20 µm ERK inhibitor PD98059 was utilized to inhibit the activation of ERK. **D** Quantification data of western blot results of rescue experiment. *n* = 3 per group (one-way ANOVA analysis with Bonferroni’s multiple comparison test). **E** Representative photography of TUNEL assay. **F** Quantification data of TUNEL assay. ERK inhibitor rescued the TAG-23 phenotype in H/R model. *n* = 3 per group (one-way ANOVA analysis with Bonferroni’s multiple comparison test). **G** Representative photography of ROS contents (up) and quantification data (down). *n* = 3 per group (one-way ANOVA analysis with Bonferroni’s multiple comparison test). **H** Representative photography of mitochondrial membrane potential in different treatment. **I** JC-1 was measured under 590/530 nm. *n* = 3 per group (one-way ANOVA analysis with Bonferroni’s multiple comparison test). **P < 0.01, ***P < 0.001. Data are means ± SD with *n* = 3 independent biological cultures

Previous studies have shown that p-ERK can protect against H/R injury by inhibiting apoptosis and oxidative stress [3, 6]. We wondered whether TAG-23 protect cardiomyocytes against reperfusion injury through ERK signaling pathway. Thus, ERK inhibitor, PD98059, was utilized to confirm the signaling pathway was inhibited. The cell damage was rescued by treatment with PD98059 and TAG-23, as indicated in cell viability and LDH release (Fig. 5A, B). Cell apoptosis was evaluated by western blot and TUNEL assay (Fig. 5C–F), which indicated that decreased apoptosis rates in TAG-23 group and increased apoptosis rates in TAG-23 and PD98059 group. Furthermore, PD98059 also restored the effect of mitochondrial membrane potential and ROS contents which was induced by TAG-23 (Fig. 5H–I). Thus, our results demonstrated that TAG-23 can bind with PKG and activate the ERK signaling pathway to attenuate apoptosis and reperfusion injury.

### TAG-23 increases the protein level of PKG by inhibiting PKG degradation

**Fig. 6:**
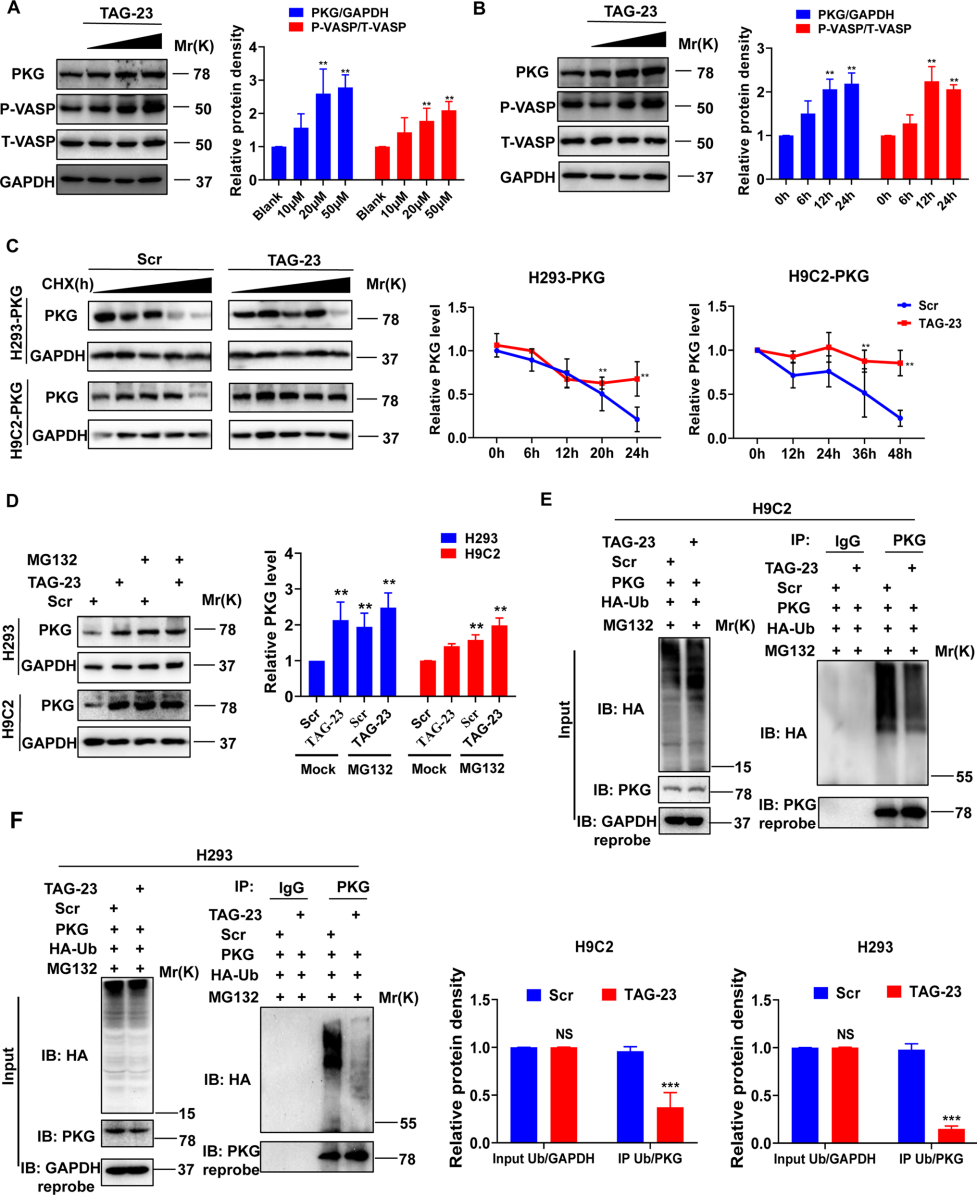
TAG-23 increases the protein level of PKG by inhibiting PKG degradation. **A** The protein level of PKG and P-VASP in different concentrations of TAG-23 (10, 20, 50 µM) in H9C2 cells. TAG-23 increased the PKG protein level in a dose-dependent manner. *n* = 3 per group (linear regression analysis). **B** The protein level of PKG and P-VASP in different time points (0, 6, 12, 24 h) in H9C2 cells. TAG-23 increased the PKG protein level in a time-dependent manner. *n* = 3 per group (linear regression analysis). **C** 1 µm CHX was used to detect the half-life of PKG in H293-PKG cells (up, 0, 6, 12, 18, 24 h) and H9C2-PKG cells (down, 0, 12, 24, 36, 48 h). TAG-23 extended the half-life of PKG in two different cell lines. Quantitative data for western blot (right). *n* = 3 per group (Student’s *t* test). **D** The protein level of PKG treated with 10 µm MG132 and 50 µm TAG-23 for 2 h. *n* = 3 per group (two-way ANOVA analysis with Bonferroni’s multiple comparison test). **E** The ubiquitination of PKG was detected in H9C2 cells. TAG-23 inhibited the PKG ubiquitination of PKG in H9C2 cells. *n* = 3 per group (Student’s *t* test). **F** The ubiquitination of PKG was detected in H293 cells. TAG-23 inhibited the PKG ubiquitination of PKG in H293 cells. *n* = 3 per group (Student’s *t* test). **P < 0.01, ***P < 0.001. Data are means ± SD with *n* = 3 independent biological replicates

To explore how the peptide TAG-23 regulates PKG in I/R, we first analyzed the mRNA level of PKG after treatment with different concentrations of TAG-23 and at different time points. There was no significant difference in the mRNA level of PKG after treatment with TAG-23, suggesting that TAG-23 had no impact on the transcription of PKG (Supple Fig S1). Interestingly, the protein level of PKG was significantly increased in H9C2 and HEK cells in a time-dependent and dose-dependent manner (Fig. 6A, B, Supple Fig. 3A, B). Besides, we also examined the P-VASP, downstream target of PKG, which was significantly upregulated upon TAG-23 treatment, suggesting that TAG-23 can activate the phosphorylation of P-VASP. These results indicated that TAG-23 may influence PKG at the posttranslational level. Previous studies have shown that PKG can be degraded through the UPS [9]. Our RNA-seq results showed that TAG-23 may be associated with Ub-mediated proteolysis (Fig. 4C); thus, we overexpressed PKG in HEK and H9C2 cells and treated them with 1 µm cycloheximide (CHX) to block protein translation [10]. TAG-23 extended the half-life of PKG in HEK cells and H9C2 cells. The protein level of PKG began to decrease at 48 h after CHX treatment, whereas TAG-23 maintained the level of PKG (Fig. 6C). We treated cells with MG132, a potent, reversible peptide aldehyde, which can effectively block the proteolytic activity of proteasome complex, to detect whether PKG degradation is influenced by the Ub system. The protein level of PKG was significantly increased after treatment with 10 µm MG132 for 2 h (Fig. 6D, lane 3 vs. lane 1). Finally, we detected whether TAG-23 can influence the ubiquitination of PKG. HA-Ub and PKG were cotransfected into H9C2 and HEK cells. After 10 µm MG132 treatment for 2 h, proteins were collected to detect ubiquitination. The total ubiquitination of proteins was unchanged. However, compared with the scramble peptide, TAG-23 significantly inhibited the ubiquitination of PKG in H9C2 cells and HEK cells after it was pulled down with PKG (Fig. 6E, F). Thus, our results demonstrated that TAG-23 can regulate the protein level of PKG at the posttranslational level by inhibiting the degradation of PKG through the UPS.

### TAG-23 inhibits PKG degradation by attenuating the PKG–cCbl interaction

**Fig. 7:**
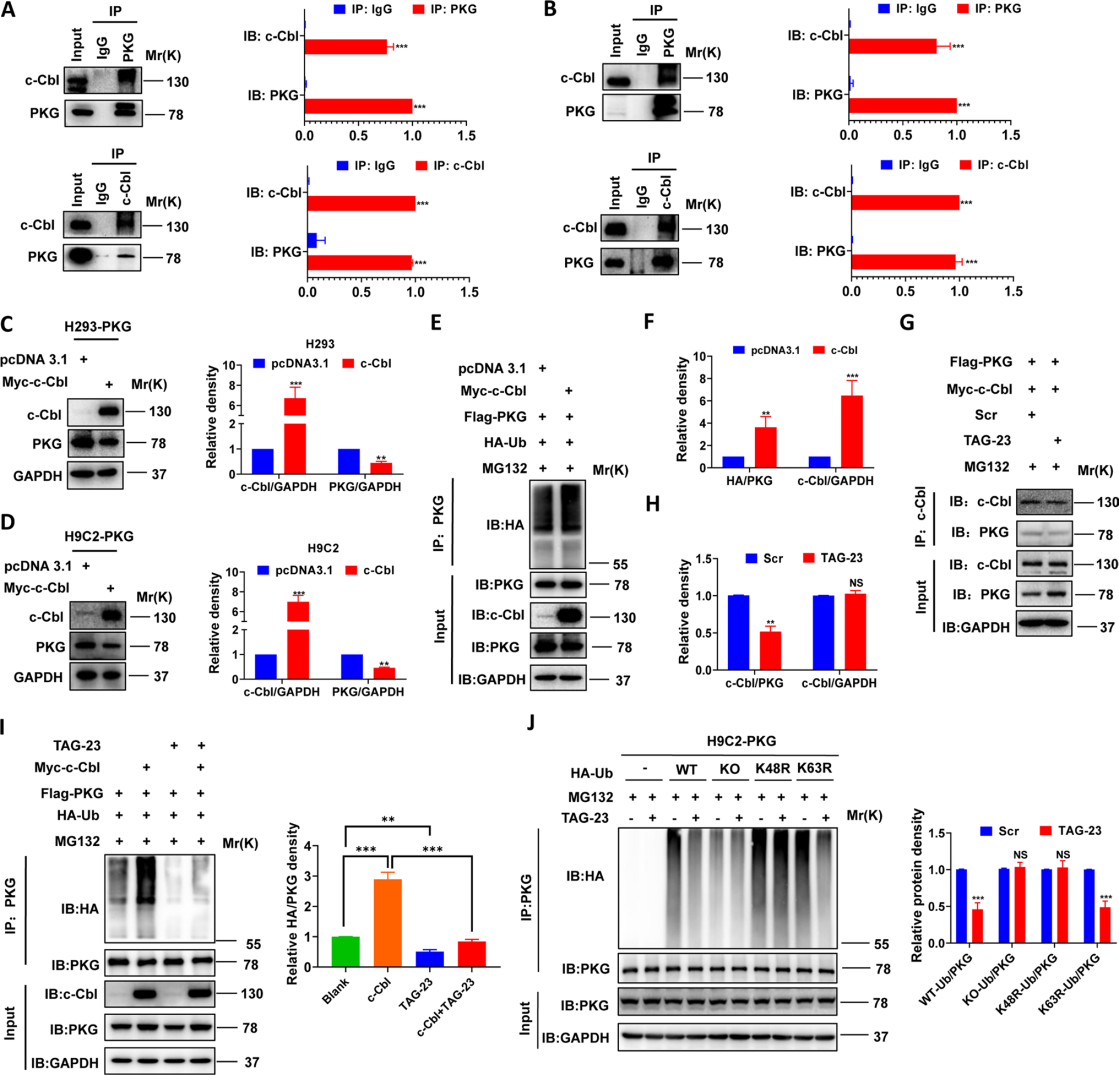
TAG-23 inhibits PKG degradation by attenuating PKG–cCbl interaction. **A** The interaction between PKG and cCbl in H293 cells was verified by Co-IP. *n* = 3 per group (Student’s *t* test). **B** PKG–cCbl interaction was verified in H9C2 cells via Co-IP. *n* = 3 per group (Student’s *t* test). **C** Overexpression of cCbl leads to the decrease of protein level of PKG in H293 cells. *n* = 3 per group (Student’s *t* test). **D** Overexpression of cCbl leads to the decrease of protein level of PKG in H9C2 cells. *n* = 3 per group (Student’s *t* test). **E** Overexpression of cCbl promotes the ubiquitination of PKG in H293 cells. Cells were treated with 10 µm MG132 for 2 h. **F** Quantification data of western blot results of ubiquitination. *n* = 3 per group (Student’s *t* test). **G** TAG-23 attenuates the PKG–cCbl interaction in H9C2 cells. TAG-23 can serve as a competitive peptide to attenuate the cCbl–PKG interaction. **H** Quantification data of western blot results of cCbl/PKG and cCbl/GAPDH. *n* = 3 per group (Student’s *t* test). **I** TAG-23 attenuates the pro-ubiquitination effect of cCbl to PKG in H9C2 cells. *n* = 3 per group (one-way ANOVA analysis with Bonferroni’s multiple comparison test). **J** TAG-23 mediated PKG degradation at the Lys48 sites in H9C2 cells. *n* = 3 per group (Student’s *t* test). **P < 0.01, ***P < 0.001. Data are means ± SD with *n* = 3 independent biological replicates

To date, there have been no reports on the E3 ligase of PKG. Therefore, we first performed a pull-down assay (Supple Fig S2). Our silver staining results showed more bands in the PKG group than the IgG group. The significant bands were incised for mass spectrometry analysis. We found that 4 E3 ligases, cCbl, RNF13, HUWE1, and PDZRN3, were enriched in the isolated bands. We cotransfected vectors overexpressing these E3 ligases and PKG in HEK293 cells. Our results showed that cCbl was pulled down by PKG in HEK cells and H9C2 cells (Fig. 7A, B). To the best of our knowledge, this is the first report on the E3 ligase of PKG. cCbl overexpression effectively decreased the protein level of PKG in HEK cells and H9C2 cells (Fig. 7C, D). To further demonstrate that cCbl is the E3 ligase of PKG, we cotransfected cCbl, PKG and HA-Ub plasmids into H293 cells. After 72 h of transfection, HEK cells were treated with 10 µm MG132 for 2 h. We found that cCbl overexpression promoted the ubiquitination of PKG (Fig. 7E, F, lane 2 vs. lane 1).

**Fig. 8:**
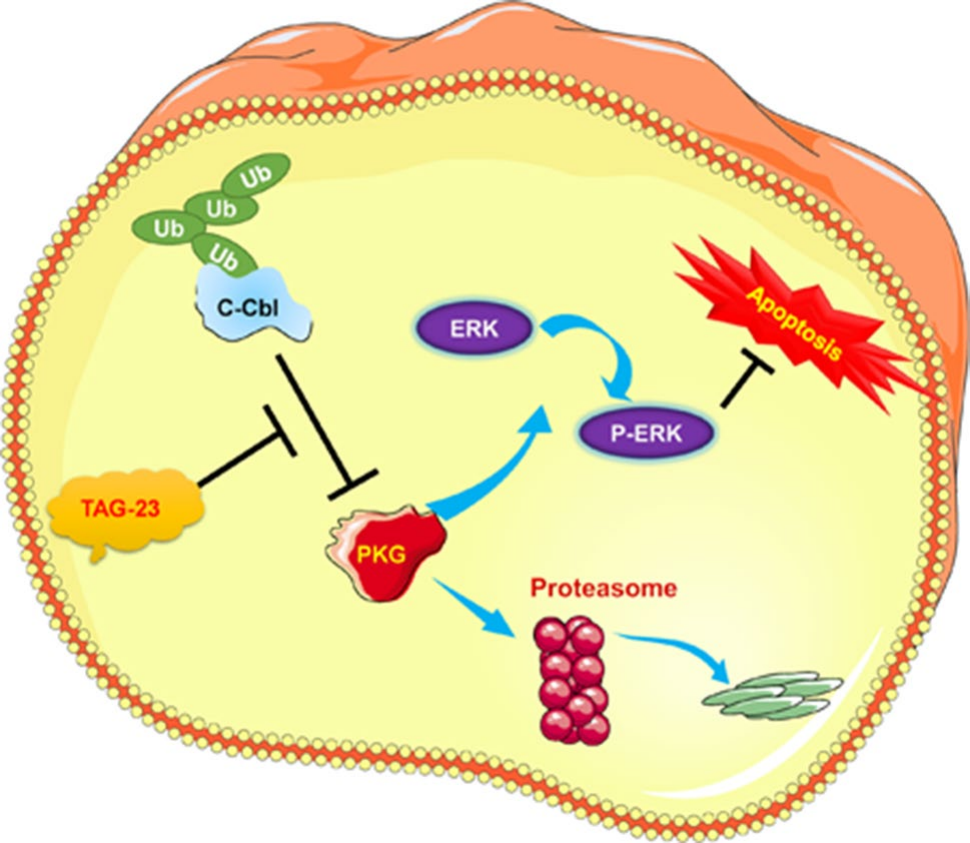
Graphic abstract TAG-23 can inhibit PKG degradation by serving as a competitive binding peptide attenuate the formation of the PKG–cCbl complex

We speculated that there may be a relationship between peptide TAG-23, cCbl and PKG. Our results demonstrated that TAG-23 treatment noticeably reduced the protein level of PKG after PKG and cCbl overexpression, suggesting that TAG-23 can serve as a competitive peptide to attenuate the PKG–cCbl interaction (Fig. 7G, H). To further verify these results, we cotransfected cCbl, PKG and Ub into H9C2 cells. After 72 h transfection, cells were treated with or without TAG-23. cCbl overexpression significantly promoted the ubiquitination of PKG (Fig. 7I, lane 2 vs. lane 1). Treatment with TAG-23 peptide reduced the ubiquitination of PKG (Fig. 7I, lane 4 vs. lane 2). Similar results can be achieved in H293 cells (Supple Fig. 3A, B). Taken together, our data provide comprehensive evidence that cCbl is the E3 ligase of PKG and that the PKG–cCbl interaction is impaired by TAG-23 treatment (Fig. 8).

### TAG-23 mediates PKG degradation at the Lys48 site

To further identify the possible ubiquitination sites, we constructed different mutant Ub plasmids (WT, KO, K48R, K63R). PKG and the mutant Ub plasmid were cotransfected into H9C2 cells. After 72 h of culture, the H9C2 cells were treated with TAG-23 and MG132 for 2 h. Our results suggested that little ubiquitination occurred without Ub overexpression (Fig. 7J, lane 2 vs. lane 1). Treatment with TAG-23 significantly inhibited ubiquitination in the WT-Ub and PKG groups (Fig. 7J, lane 3 vs. lane 4). However, there was no difference in the KO group with or without TAG-23 treatment (Fig. 7J, lane 6 vs. lane 5). Interestingly, there was still a significant difference in the K63R mutant Ub group, suggesting that the K63R mutant had no impact on the ubiquitination of PKG (Fig. 7J, lane 9 vs. lane 10). We also verified above results in H293 cells (Supple Fig. 3E, F). These effects suggested that TAG-23 mediates PKG degradation at the Lys48 site.

## Discussion

Here, we demonstrate that peptide TAG-23 has a therapeutic role in reperfusion and oxidative stress, which result in MI and heart failure. First, TAG-23 peptide was shown to be efficiently internalized in cardiomyocytes and reduce cell apoptosis, ROS content, and oxidative stress in vitro. In vivo, TAG-23 peptide significantly reduced the infarct size in a MI model. Moreover, we demonstrated that TAG-23 can inhibit PKG ubiquitination by attenuating the interaction between PKG and cCbl. TAG-23 peptide appears to be a valuable therapeutic candidate because the crucial role of PKG and ERK in I/R injury. Our study provides evidence that peptide TAG-23 is a promising candidate for MI therapy.

To explore the mechanism underlying the effect of TAG-23 peptide in reperfusion injury, we first performed a pull-down assay to identify the potential binding target of TAG-23. A silver staining assay showed 2 different bands, and a total of 235 proteins that may interact with TAG-23 were identified by mass spectrometry. Among these proteins, PKG was the most highly expressed. Further coimmunoprecipitation (Co-IP) assays confirmed the interaction between TAG-23 and PKG in HEK293 and H9C2 cells. However, due to the lack of a TAG-23 antibody, we failed to verify that PKG can be pulled down by TAG-23. Cyclic GMP-dependent protein kinase (PKG) belongs to the serine/threonine kinase family and is an important mediator of nitric oxide (NO) [14]. Previous studies have reported that PKGIα is mainly expressed in cardiomyocytes [11]. It is interesting to note that PKG, an important regulator of cardioprotection, activates ERK [7], which plays prosurvival roles, and inhibits JNK [6] and p38 kinase [11], which exert proapoptotic effects on cardiovascular diseases [38, 15]. DOX is a frequently used chemotherapeutic agent used to treat numerous tumors [19], and it can also cause irreversible cardiac toxicity, including heart failure [40], cardiomyocyte apoptosis [17], and oxidative stress [20]. Although numerous studies have focused on DOX-induced heart failure, the underlying mechanism remains unclear [22]. In our study, we found that H/R and DOX treatment significantly reduced the protein level of phosphorylated ERK and that the TAG-23 peptide increased the protein level of p-ERK. However, TAG-23 had no effect on the protein level of p-p38 or p-JNK. We speculated that TAG-23 protects cardiomyocytes against reperfusion and oxidative stress by activating the PKG-ERK signaling pathway. Thus, the downstream ERK activation is upregulated upon TAG-23 treatment.

Previous studies have shown that the half-life of PKG can be very long or rather short depending on the cell type [29]. For example, the half-life of PKG is approximately 24 h in vascular smooth muscle cells (VSMCs) and 60 days in platelets. However, there have been no reports regarding the specific half-life of PKG in cardiomyocytes. We found that the half-life of PKG was extended to varying degrees in cardiomyocytes and HEK293 cells. In smooth muscle cells, PKG can be degraded through the ubiquitin (Ub)/26S proteasome system (UPS), and mutation of the Ser64 autophosphorylation site to an alanine residue can block the degradation of PKG [9]. Another study showed that Ub molecules can easily bind to PKG-I under hypoxic conditions, leading to accumulation of ubiquitinated PKG-I [26]. PKG can positively regulate proteasome activities, and activation of PKG by sildenafil can reduce the accumulation of misfolded proteins by stimulating the UPS in cardiomyocytes [25, 28]. It is well established that E3 ligase is responsible for targeting specific substrate proteins for ubiquitination. Although the function and downstream genes of PKG have been extensively investigated, there have been no reports on the E3 ligase of PKG, and how PKG is regulated in I/R injury remains unknown. In our study, we identified that the E3 ligase cCbl can bind with PKG and promote its degradation. Thus, we overexpressed cCbl and PKG in H9C2 and HEK293 cells to verify the influence of TAG-23 on ubiquitination. Our results demonstrated that TAG-23 attenuated the interaction between PKG and cCbl, thus reducing the degradation of PKG and alleviating cell apoptosis. However, ubiquitin possesses several different lysine residues that serve as ubiquitination sites, of which Lys48 and Lys63 are the most well characterized. As expected, mutation of the Lys48 site to an arginine residue almost completely abolished TAG-23-mediated PKG degradation, whereas there was no significant difference in PKG degradation in the presence of the K63R mutation. These data show that TAG-23 mediates PKG polyubiquitination degradation at the Lys48 site. Previous studies have also shown that autophosphorylation of Ser64 can trigger the downregulation of PKG [9, 29]. Thus, further studies that identify the binding site between TAG-23 and PKG and determine whether Ser64 mutation can block the function of TAG-23 are warranted.

As previously reported, four healthy male volunteers were undergoing cycle exercise for 6 min at 77% of individual Wmax and then to exhaustion at 87–88% of Wmax. TAG-23 was induced by exercise and TAG-23 was increased 2.659-fold change after exercise. Despite the endogenous peptide TAG-23 content rose about 2.659-fold after the exercise, the amount of TAG-23 induced by exercise was still limited. Nonetheless, the exogenous supplement of TAG-23 in vivo and in vitro achieved great beneficial effects. Another example is recombinant human brain natriuretic peptide. BNP level in healthy people is much lower than patients with heart failure. However, recombinant human BNP can successful applied for heart failure patients. In addition, the exercise-induced peptidome has also enable the identification of new peptides that may possess roles in different physiological processes and organ crosstalk. For example, 22 peptides derived from kininogen-1 (KNG) are significantly increased during exercise. Peptides are typically derived from the degradation of proteins, executed by the UPS system. Commonly, the protein degradation process regulated by UPS was radically and we speculated that a particular protease or peptidase might have been activated during the exercise process, which digested TAGLN and generated peptide TAG-23. Exploring the regulatory network may be an intriguing field to follow in the future. Taken together, these findings confirm that beneficial effects are mediated by peptides and other circulating factors. It is possible that several secreted peptides may exert beneficial effects, at least in part, during exercise.

Previous studies have suggested that transgelin interacts with actin stress fibers and stabilizes actin in vivo [18]. Transgelin has also been shown to be associated with a specific subpopulation of actin filament bundles that form podosomes [31]. The percentage of infarction (infarction ratio) for wild-type MI mice was measured as 11.2% while that for transgelin(− / −) mice was measured as 16.2%. SAA (A traditional Chinese medicine) could stabilize the transgelin-actin complex, modulate the reorganization of the actin cytoskeleton, facilitate F-actin bundling, further enhance the contractility and blood flows of coronary arteries, and reduce infarct size. In addition to be a fundamental component of the cytoskeleton, actin has been found to carry out different functions in the cell nucleus [32]. Actin has been shown to be associated with chromatin remodeling and RNA processing [27, 32]. In our study, we found that the peptide TAG-23, which is derived from the structural protein transgelin, can alleviate cell apoptosis and MI. Structural proteins, which constitute the cell structure and allow mobility, are considered components of the cytoskeleton [30]. Previous studies have confirmed that the rapid and specific binding of Smad3 to the transgelin promoter. TGF-β treatment resulted in rapid up-regulation of transgelin in A549 cells. Transgelin expression was specifically localized to ATII and smooth muscle cells in mouse lungs, and its expression was augmented in ATII cells in lung fibrosis. Our results may provide a certain reference value for the function of transgelin protein to reduce the area of myocardial infarction.

Our study still has some limitations. First, for further clinical application, a detailed evaluation of response time in murine models or clinical animal models is essential to fully understand the time window of cardioprotection in MI. Second, the relationship between peptide TAG-23 and TGF-β in cardiomyocytes remain elusive. Exploring the regulatory work in cardiomyocytes will further explain the effect of TAG-23.

In summary, we performed a comprehensive functional analysis of the peptide TAG-23, which can inhibit cell apoptosis and oxidative stress in vitro and ameliorate MI and heart failure in vivo. We also showed that targeting the PKG–cCbl interaction with an endogenous pharmacological peptide directly reduced the degradation of PKG, thus allowing activation of its downstream protein ERK. Our study provides a new approach for treating MI.

## Supplementary Information

Below is the link to the electronic supplementary material.

Supplementary file1 (TIFF 293 KB)

Supplementary file2 (TIFF 431 KB)

Supplementary file3 (PDF 74 KB)

Supplementary file4 (XLSX 9 KB)

Supplementary file5 (XLSX 10 KB)

Supplementary file6 (XLSX 13 KB)

Supplementary file7 (XLSX 13 KB)

Supplementary file8 (XLSX 13 KB)

Supplementary file9 (XLSX 15 KB)

Supplementary file10 (PDF 380 KB)

Supplementary file11 (XLSX 14 KB)

Supplementary file12 (XLS 20 KB)

Supplementary file13 (PDF 174 KB)

Supplementary file14 (PDF 570 KB)

Supplementary file15 (PDF 819 KB)

Supplementary file16 (PDF 526 KB)

## Data Availability

Original data of RNA-sequencing and mass-spec submitted with the manuscript. Data associated with temporal quantification of the exercise-regulated peptidome are available in project accession PXD004781 (username: reviewer61660@ebi.ac.uk; password: NaHrEPqj).

## Abbreviations

LDH: Lactate dehydrogenase
ROS: Reactive oxygen species
DOX: Doxorubicin
PKG: Cyclic GMP-dependent protein kinase
NO: Nitric oxide
CHX: Cycloheximide
ERK: Extracellular signal-related kinases
JNK: Jun amino-terminal kinases
UPS: Ubiquitin proteasome system
LVEDd: Left ventricular end-diastolic dimensions
LVPWd: Left ventricular posterior wall dimensions
LVPWs: Left ventricular posterior wall at end systole
EF: Ejection fraction
FS: Fractional shortening
LVEDs: Left ventricular end-diastolic stiffness
PBS: Phosphate-buffered saline
