# Supplementary figures and images for: Exercise-induced peptide TAG-23 protects cardiomyocytes from reperfusion injury through regulating PKG–cCbl interaction
Source: Basic Res Cardiol. 2021 Jun 25;116(1):41. doi: 10.1007/s00395-021-00878-4 (PMC8233271; doi:10.1007/s00395-021-00878-4)

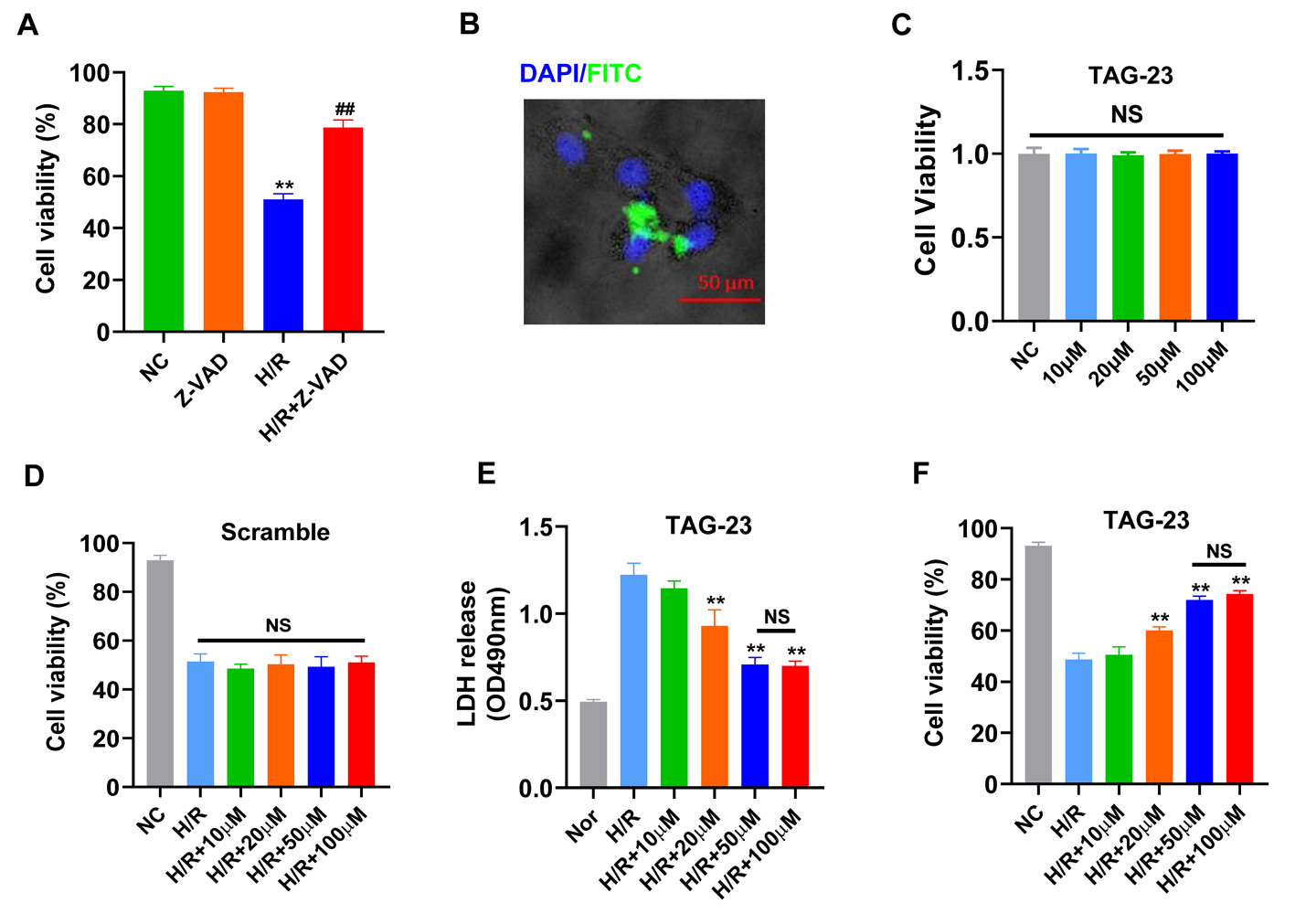

Supplement: Supplementary file 1 — Supplementary file1 (TIFF 293 KB) [file 395_2021_878_MOESM1_ESM.tiff]

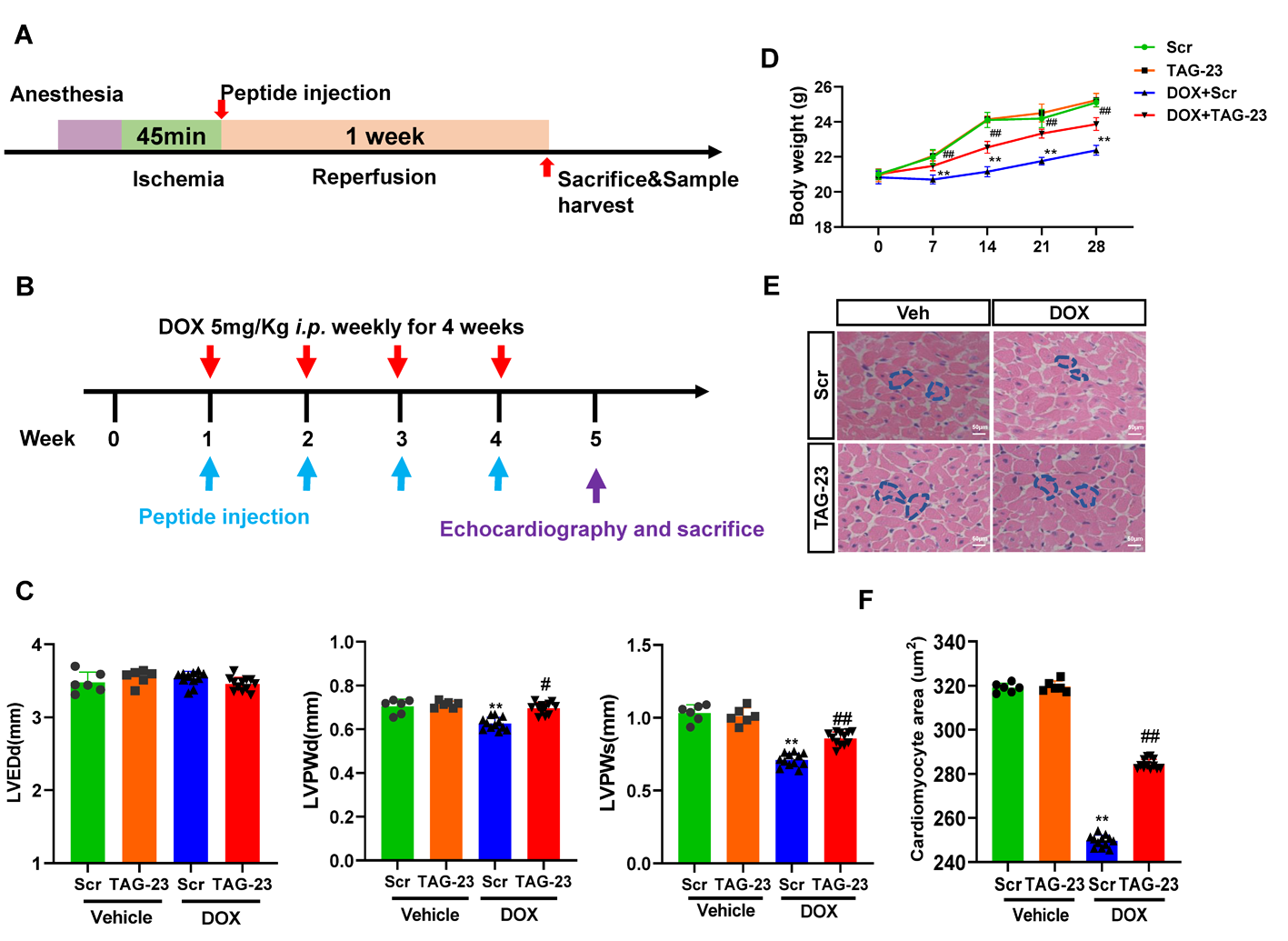

Supplement: Supplementary file 2 — Supplementary file2 (TIFF 431 KB) [file 395_2021_878_MOESM2_ESM.tiff]

Figure 1C

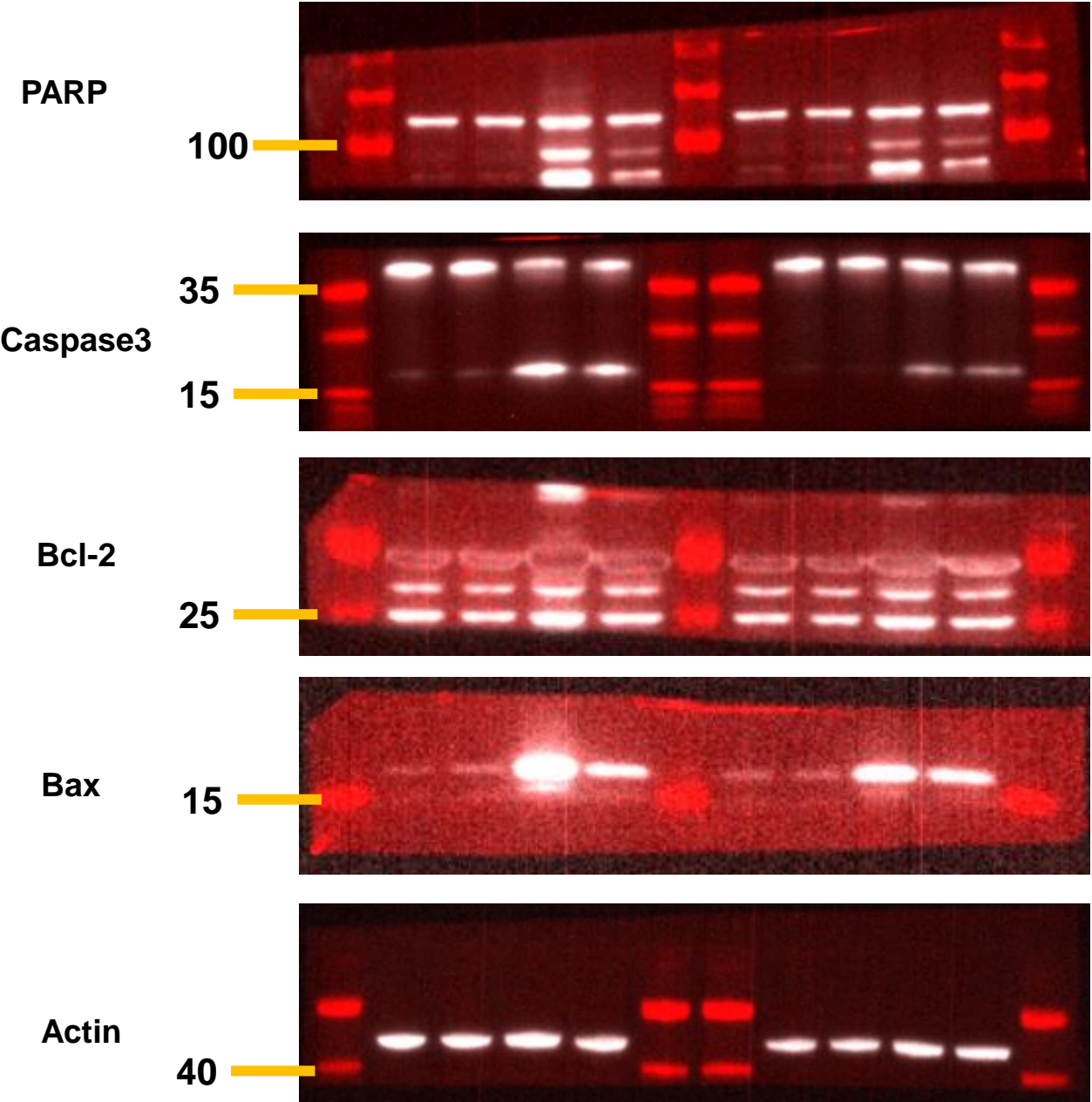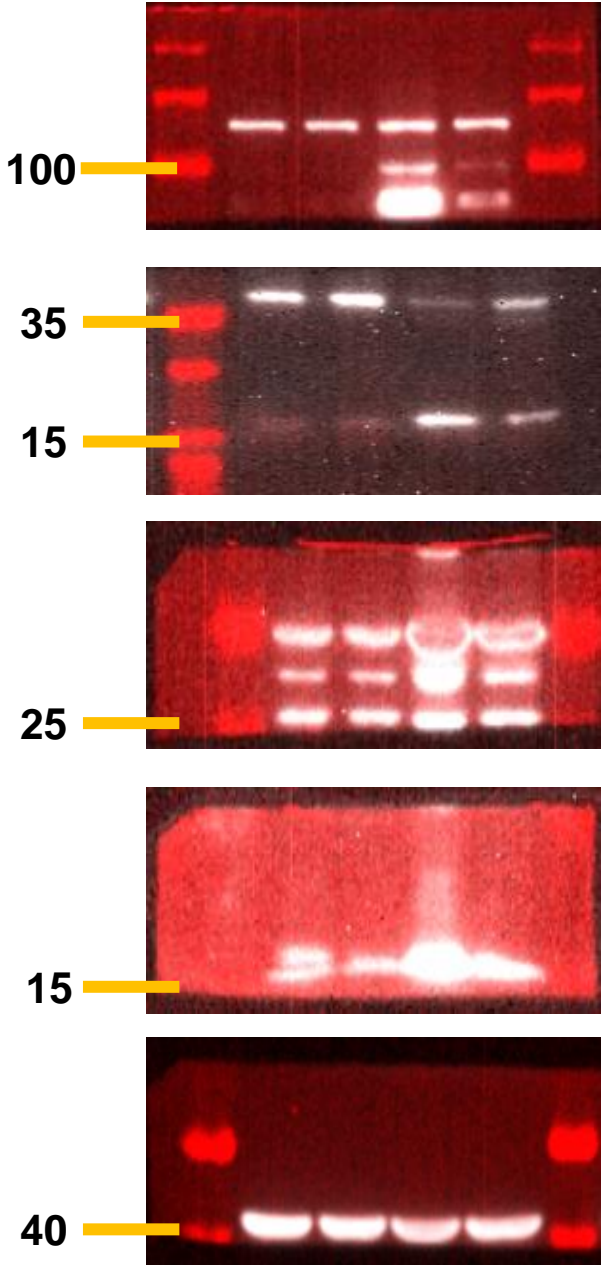

Supplement: Supplementary file 3 — Supplementary file3 (PDF 74 KB) [file 395_2021_878_MOESM3_ESM.pdf]

**Figure 4D**

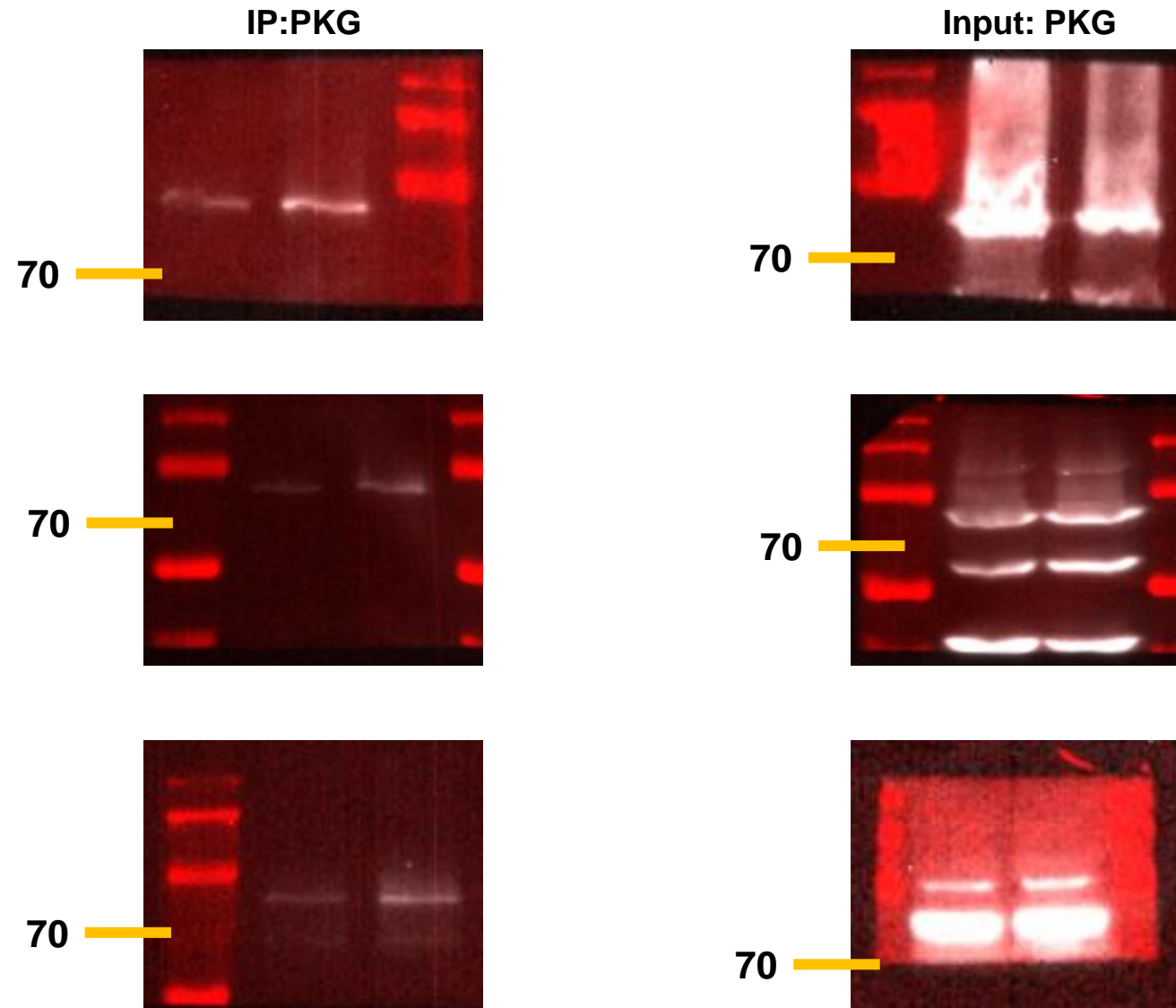

**Figure 4D**

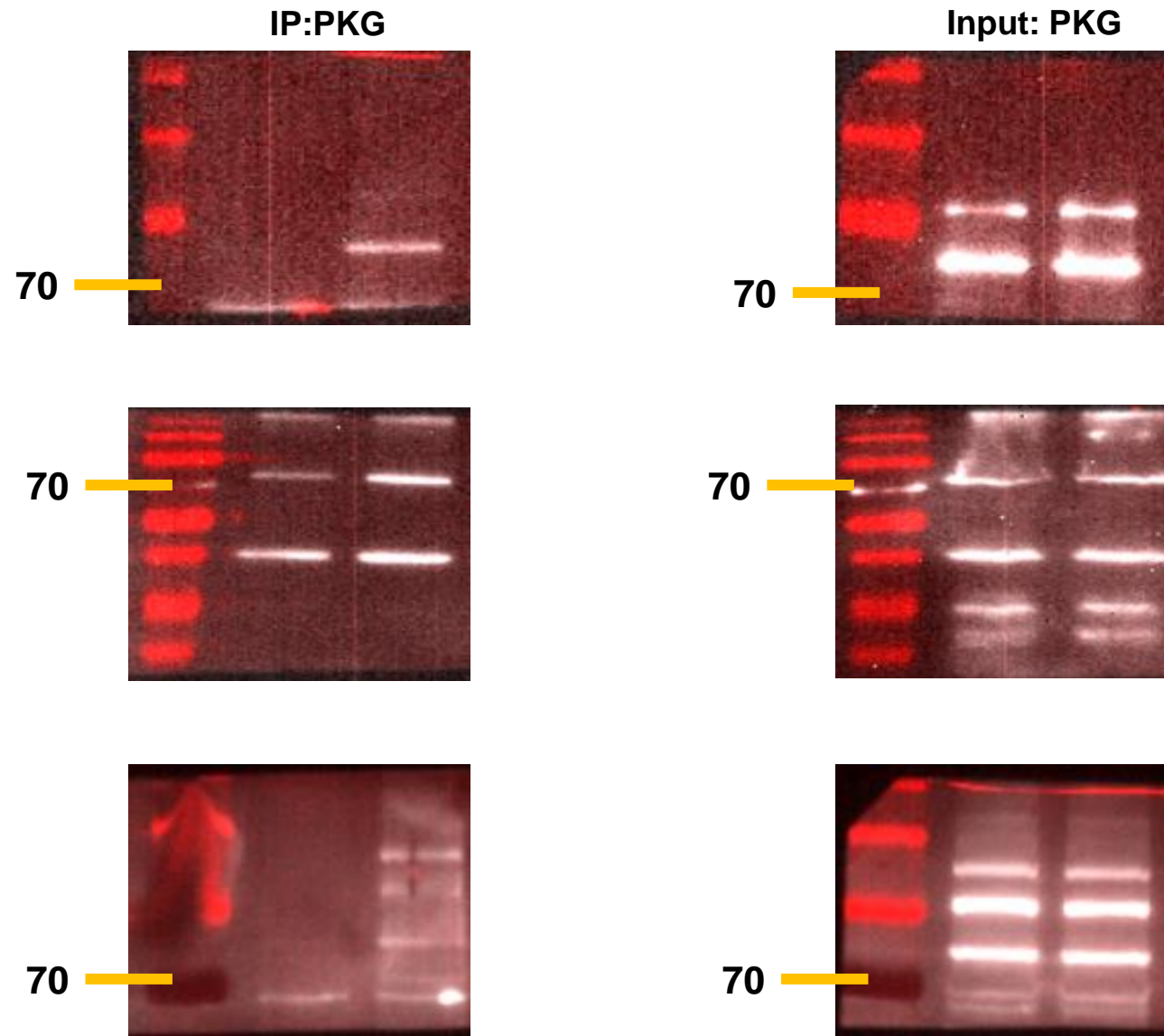

**Figure 4E**

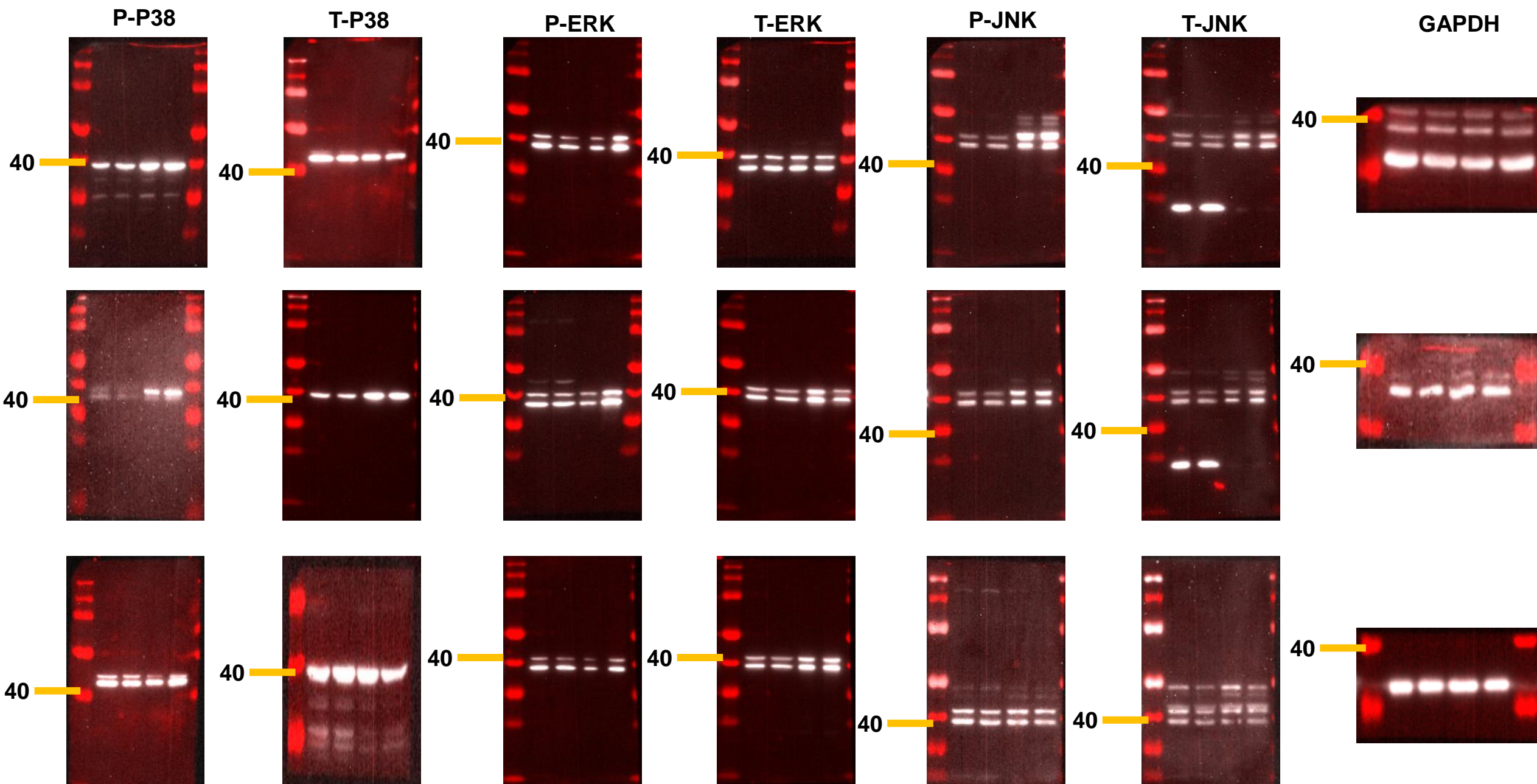

## Figure 4F

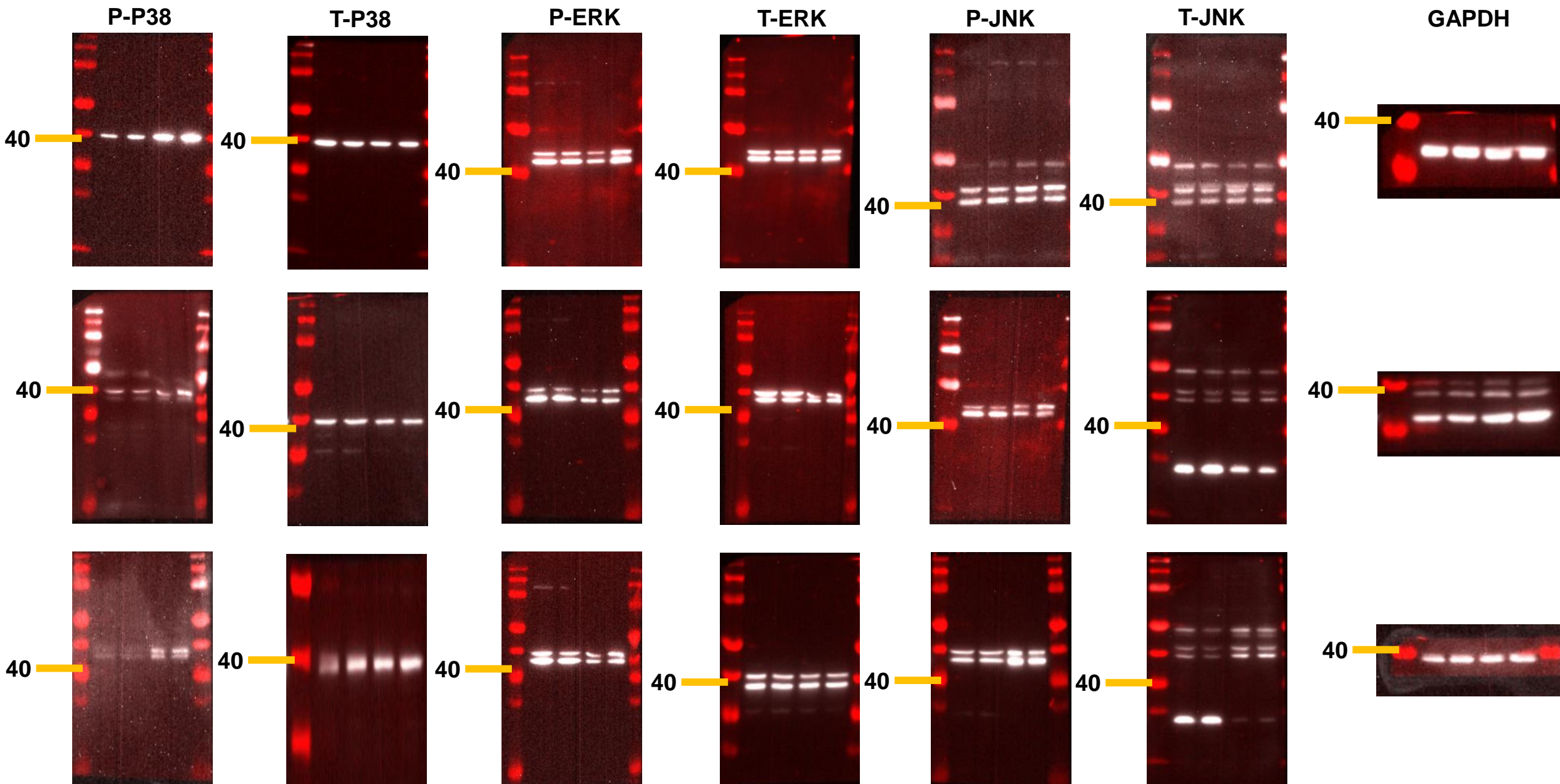

Supplement: Supplementary file 10 — Supplementary file10 (PDF 380 KB) [file 395_2021_878_MOESM10_ESM.pdf]

**Figure 5C**

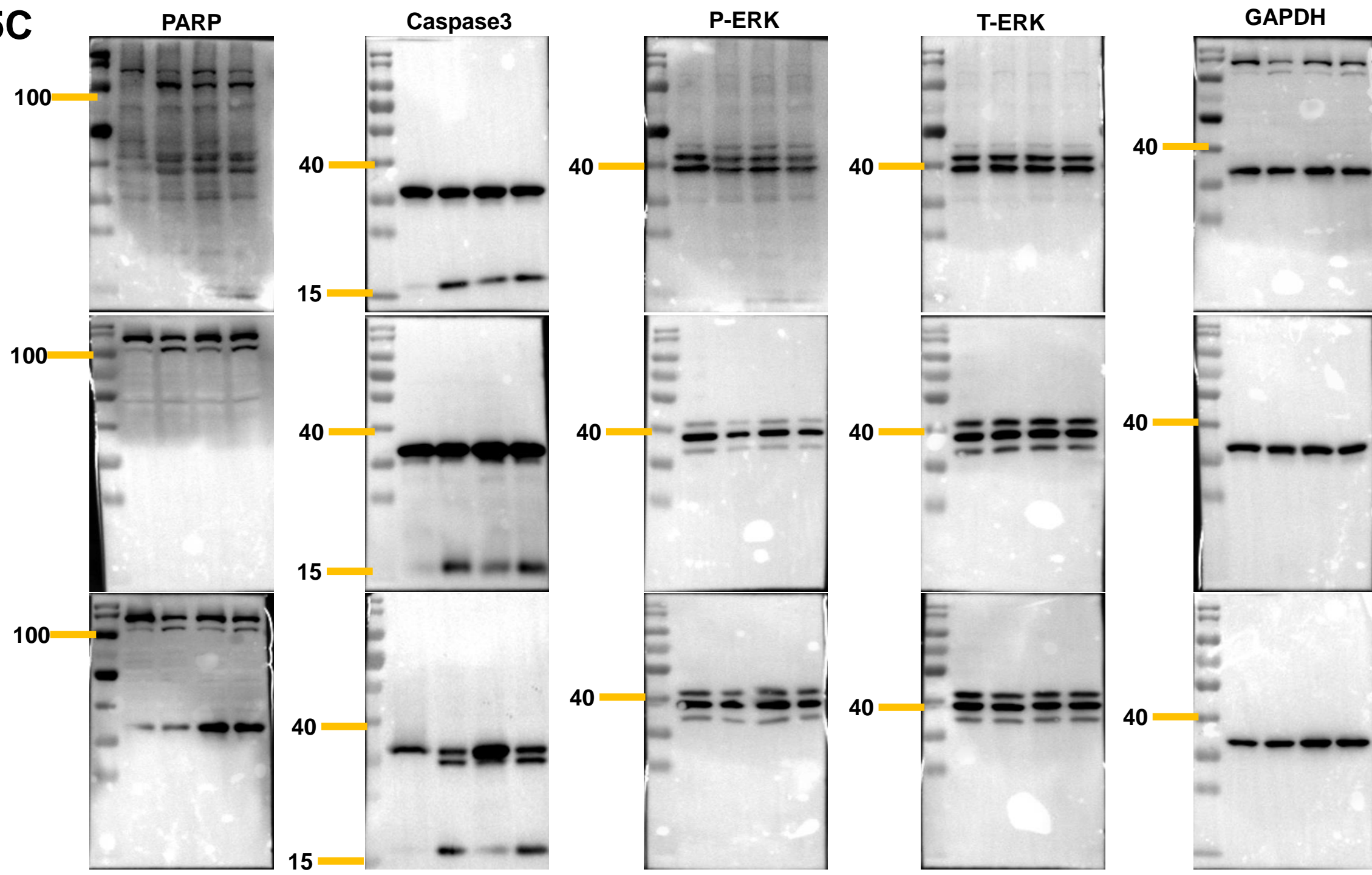

Supplement: Supplementary file 13 — Supplementary file13 (PDF 174 KB) [file 395_2021_878_MOESM13_ESM.pdf]

**Figure 7A**

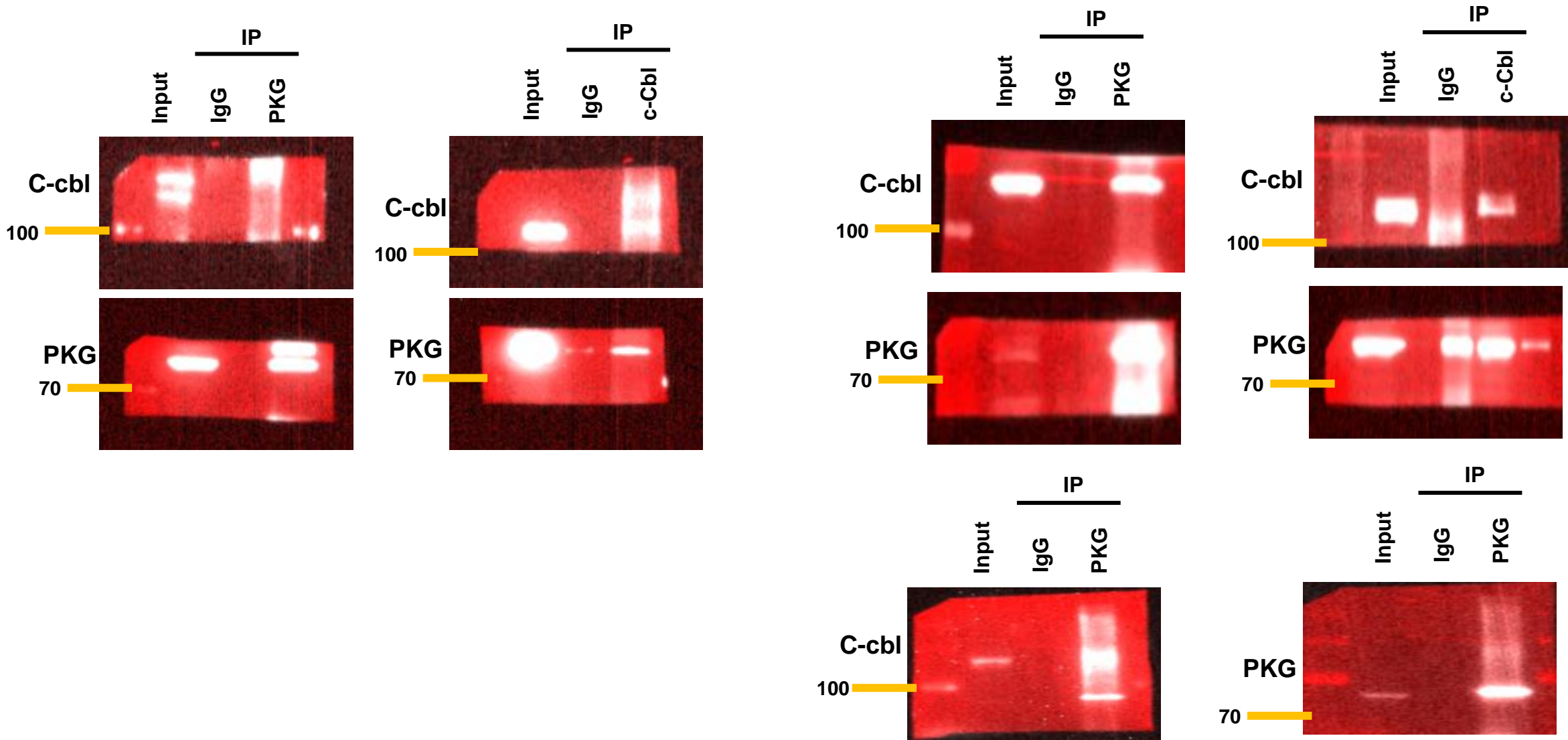

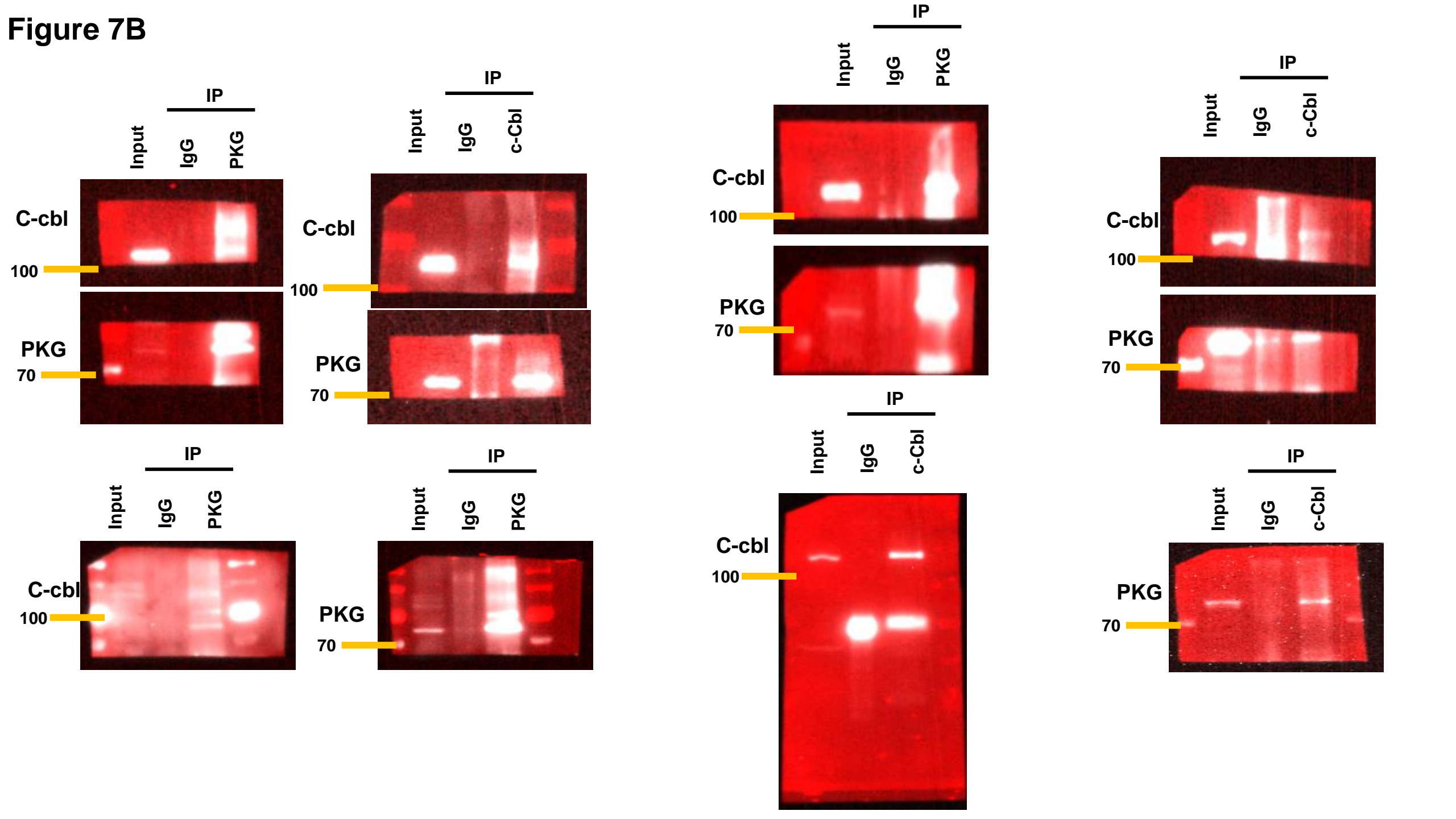

**Figure 7C H293**

**C-cbl**

100

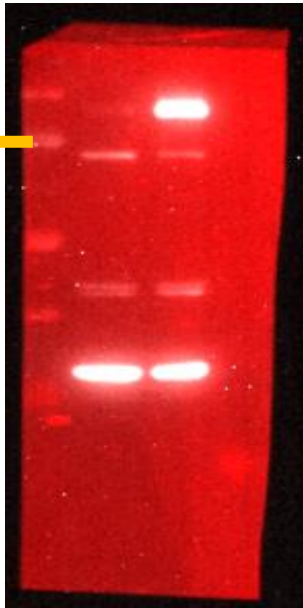

**PKG**

70

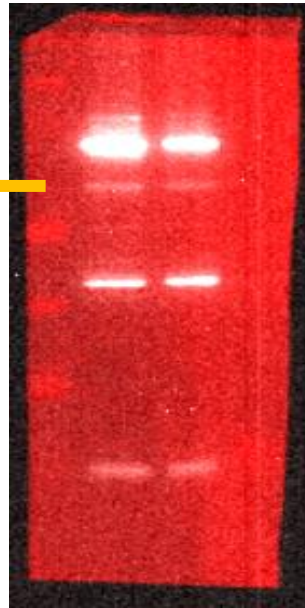

40

**GAPDH**

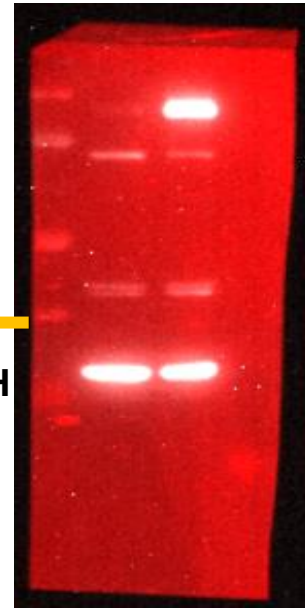

**C-cbl**

100

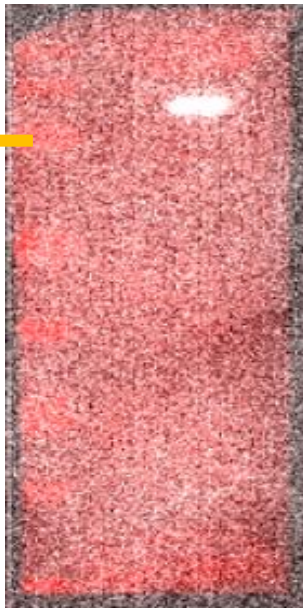

**PKG**

70

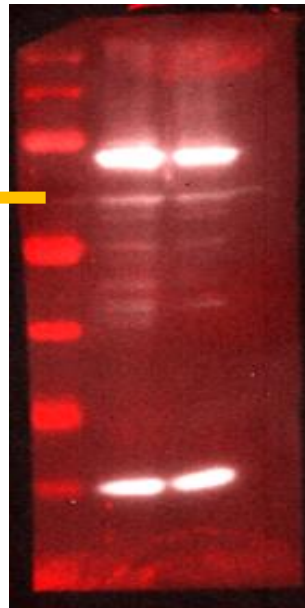

40

**GAPDH**

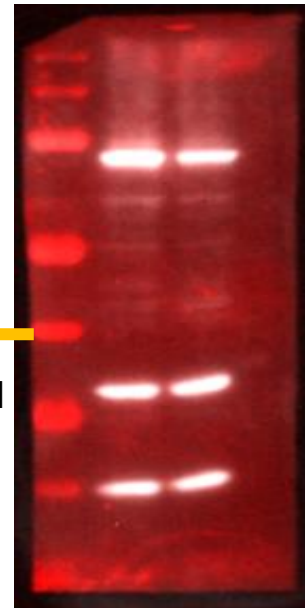

**Figure 7D H9C2**

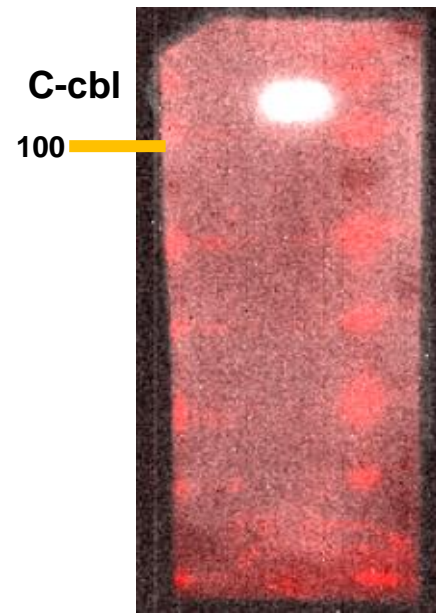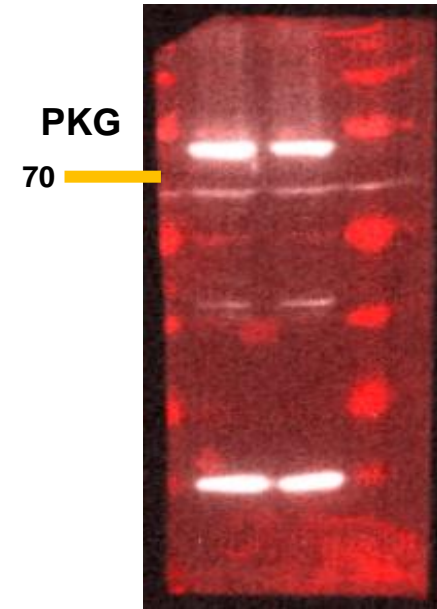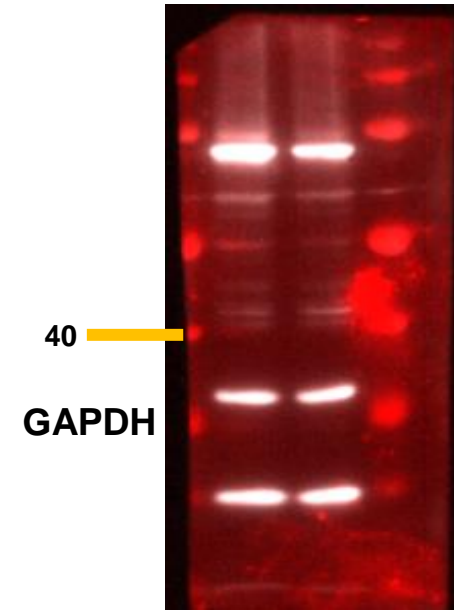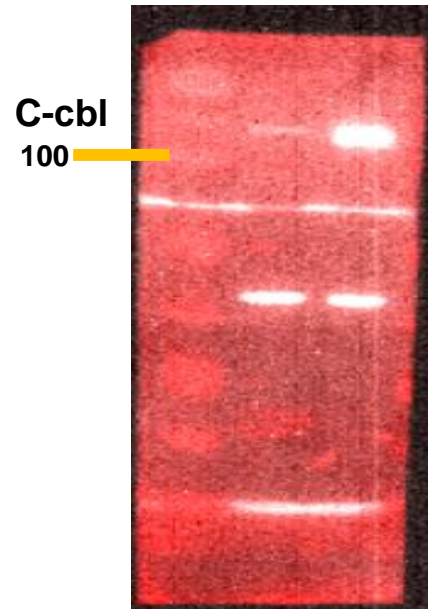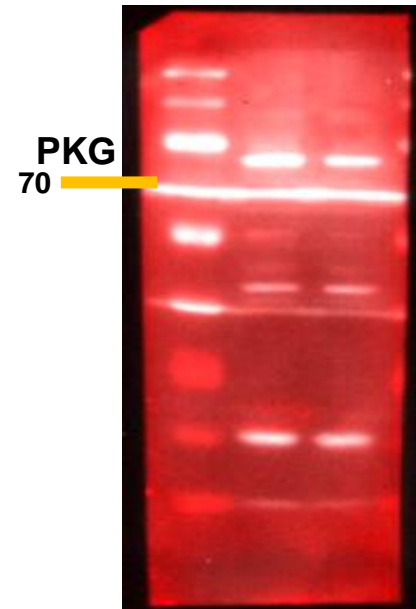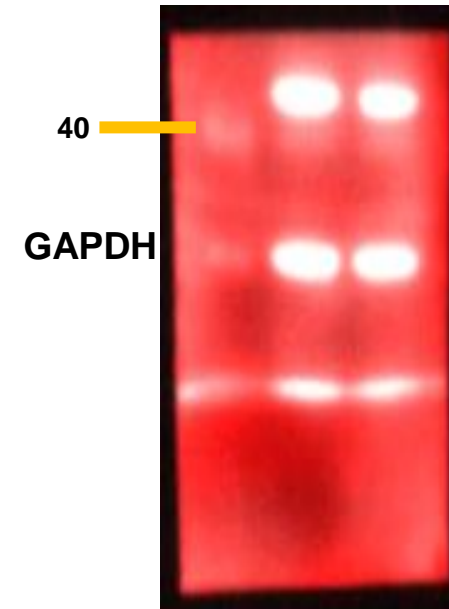

**Figure 7E**

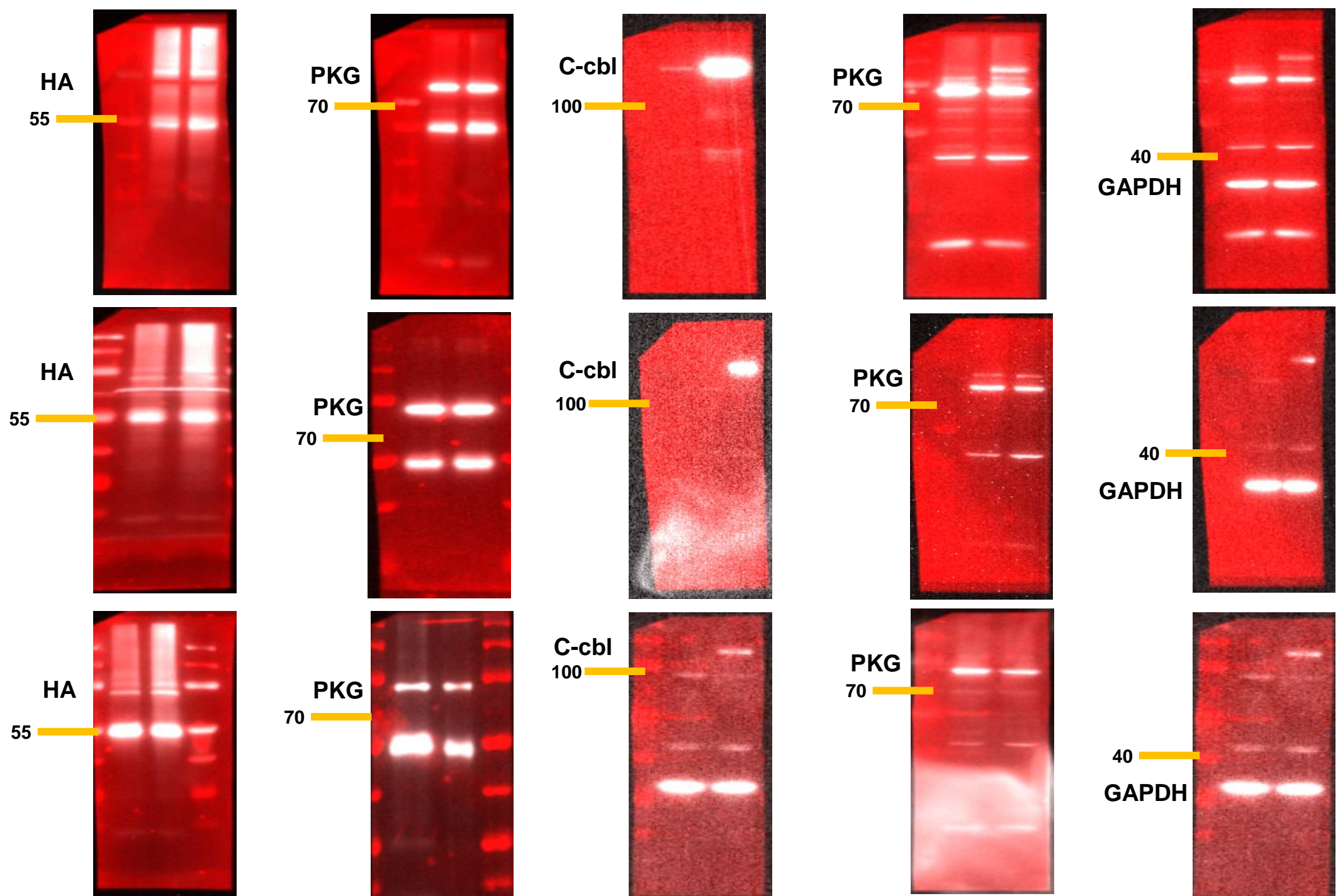

**Figure 7G**

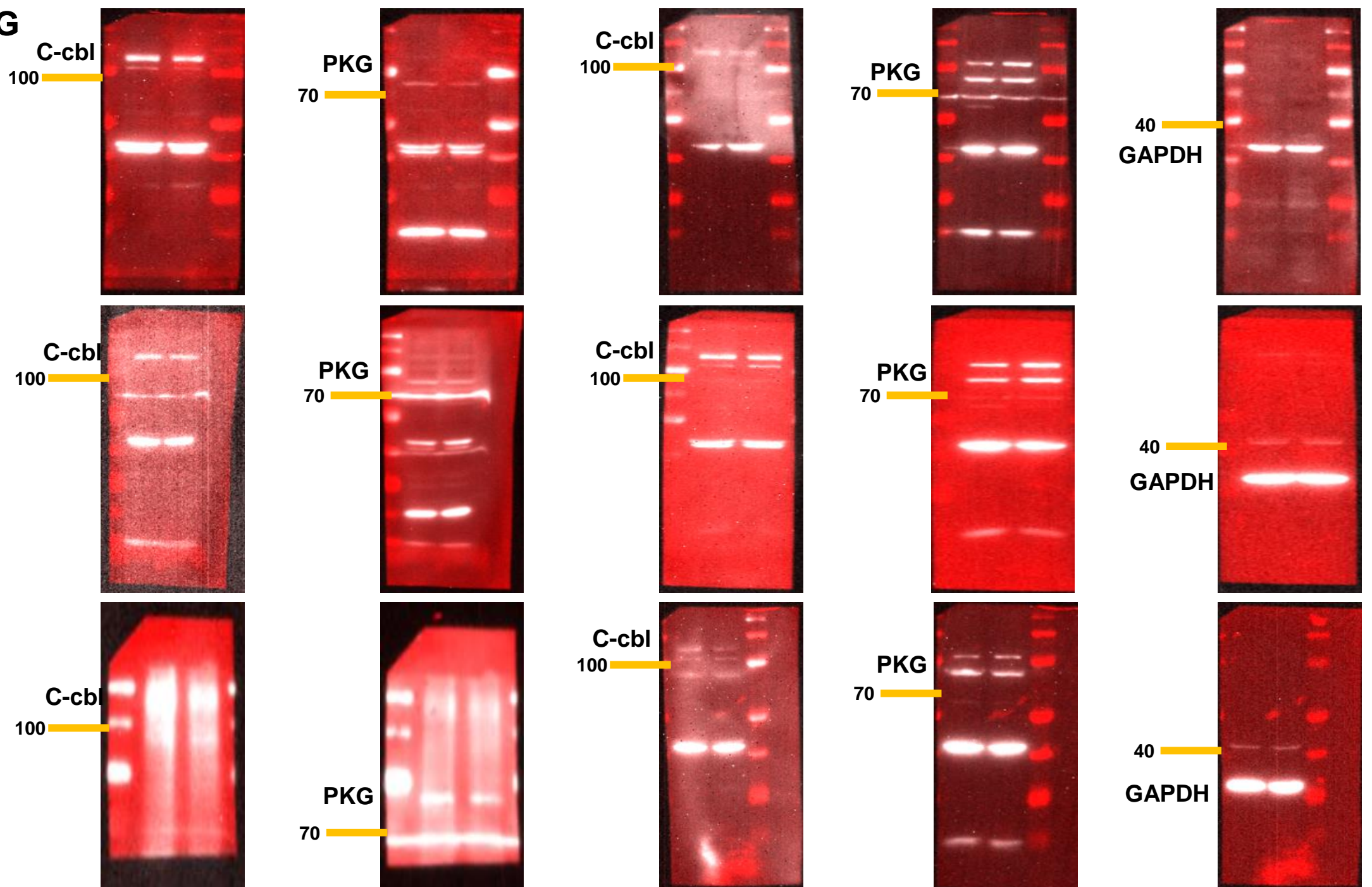

**Figure 7I**

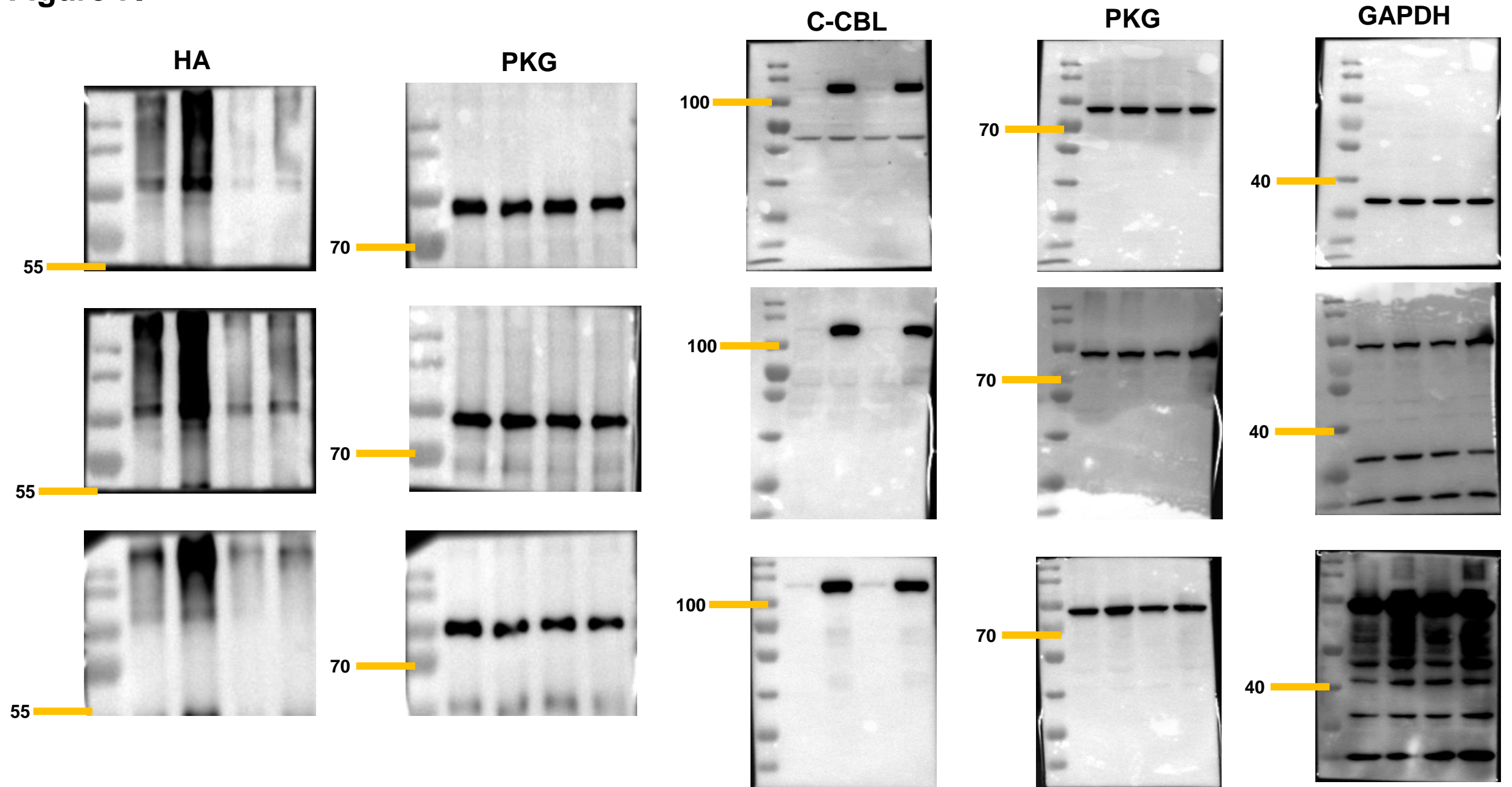

**Figure 7J**

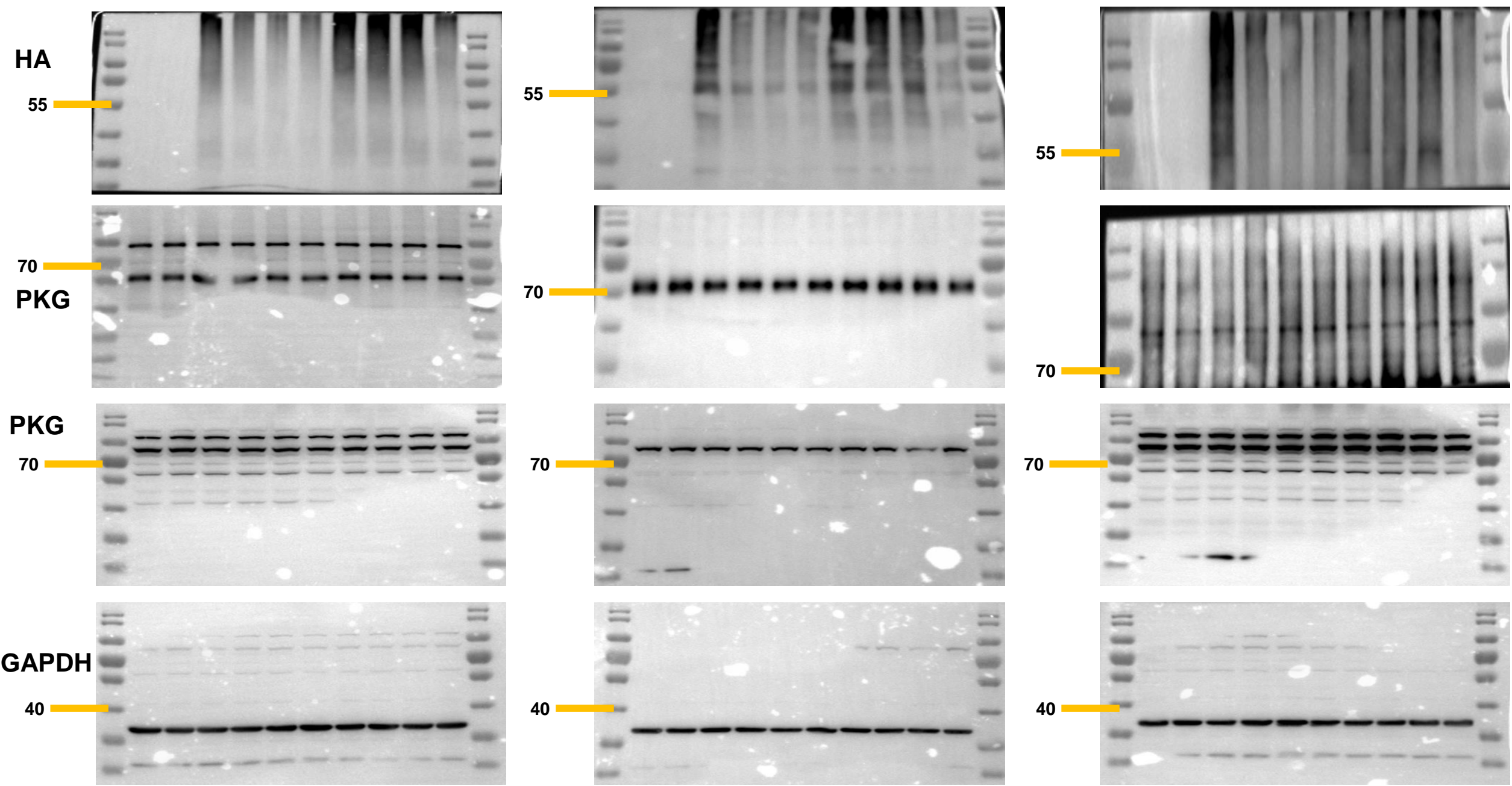

Supplement: Supplementary file 15 — Supplementary file15 (PDF 819 KB) [file 395_2021_878_MOESM15_ESM.pdf]

# Supplemental Figure 3A

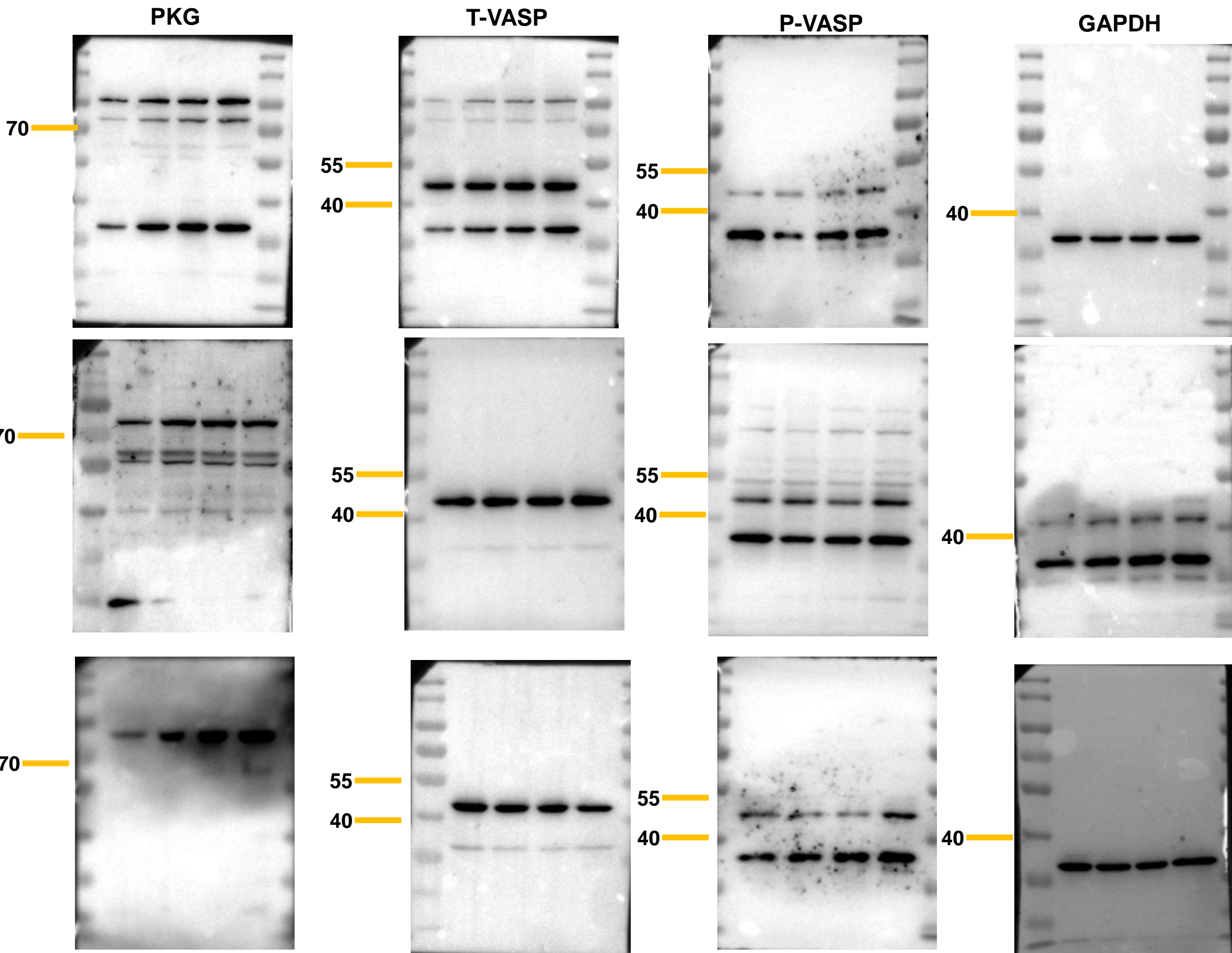

# Supplemental Figure 3B

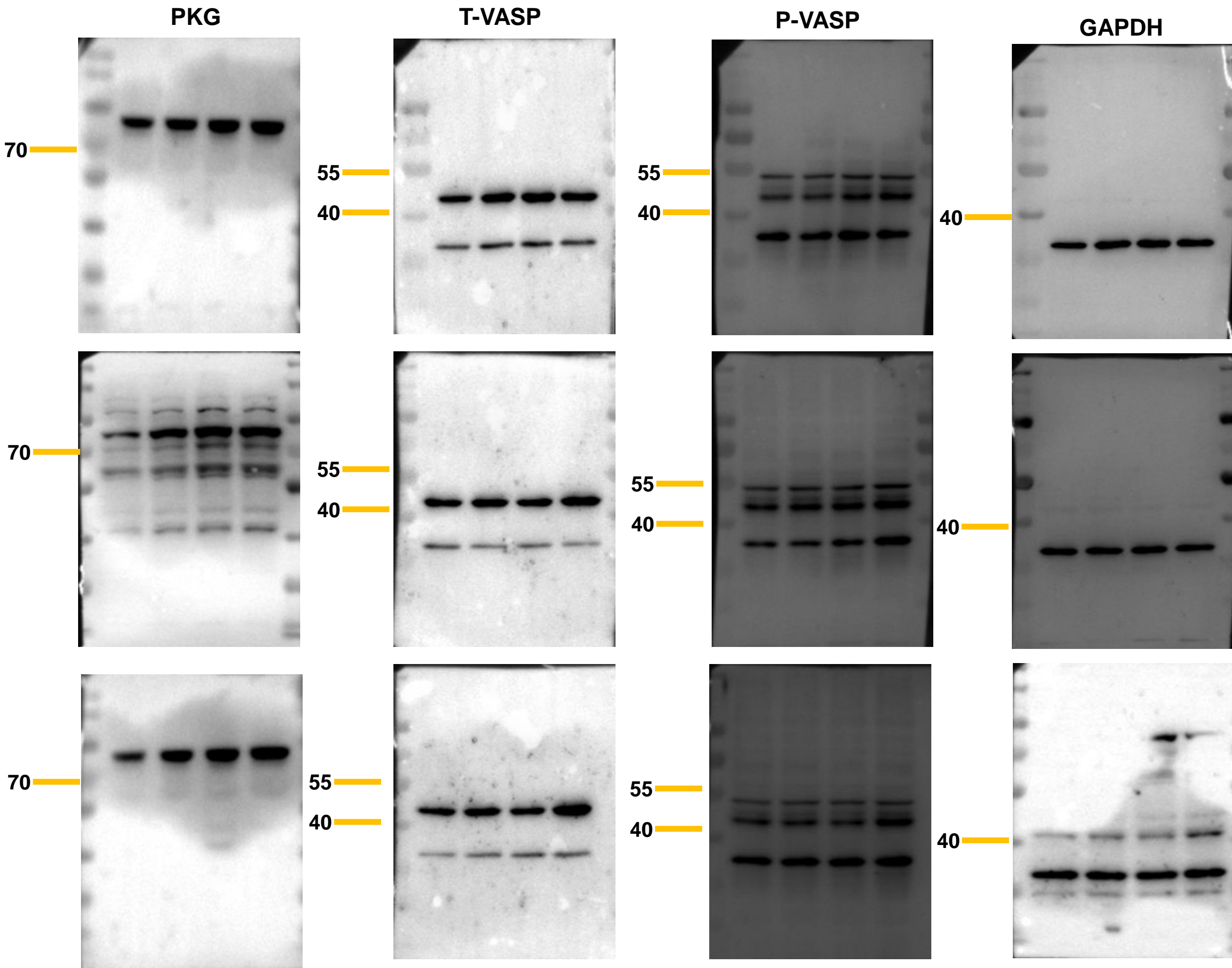

# Supplemental Figure 3C

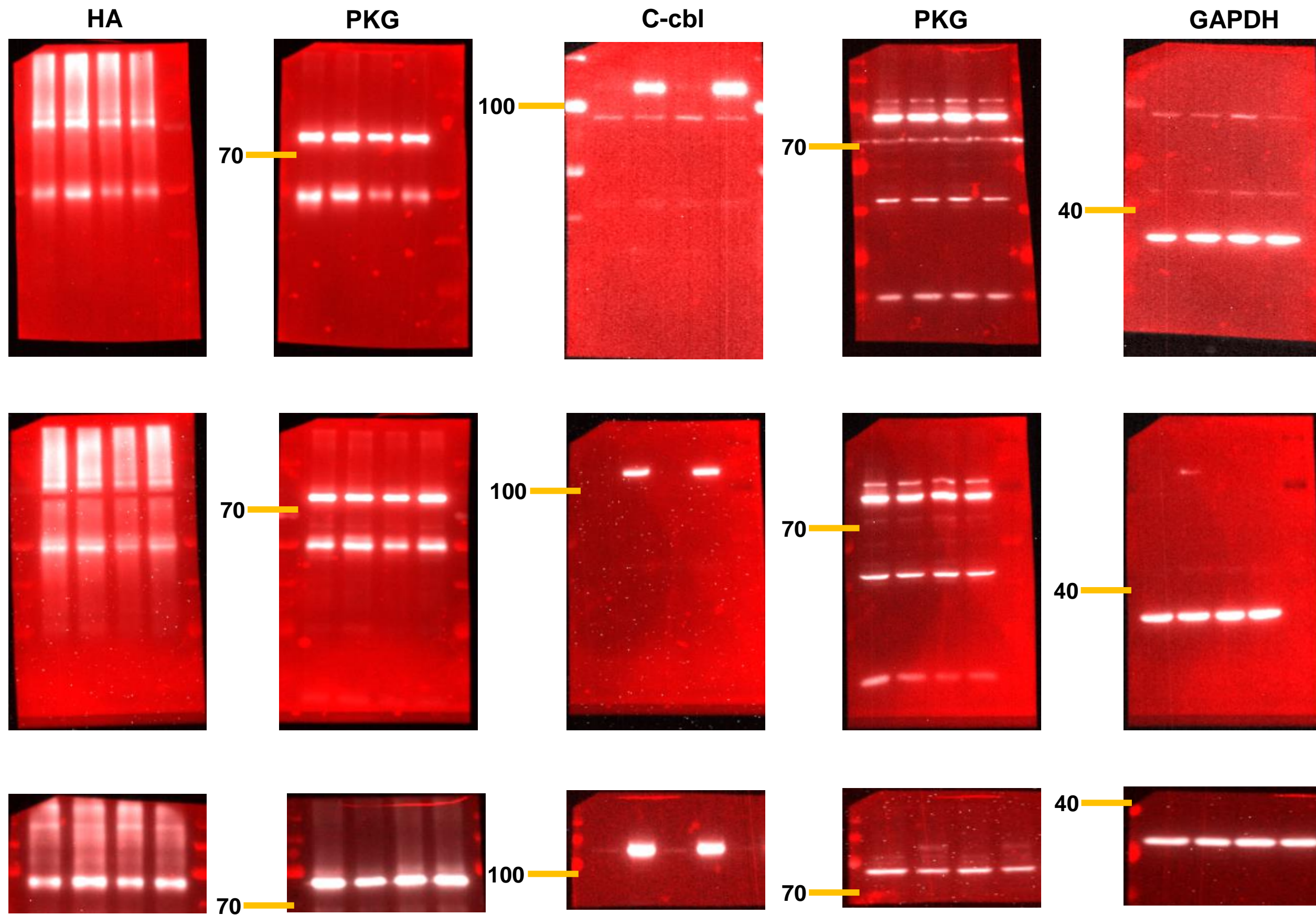

# Supplemental Figure 3D

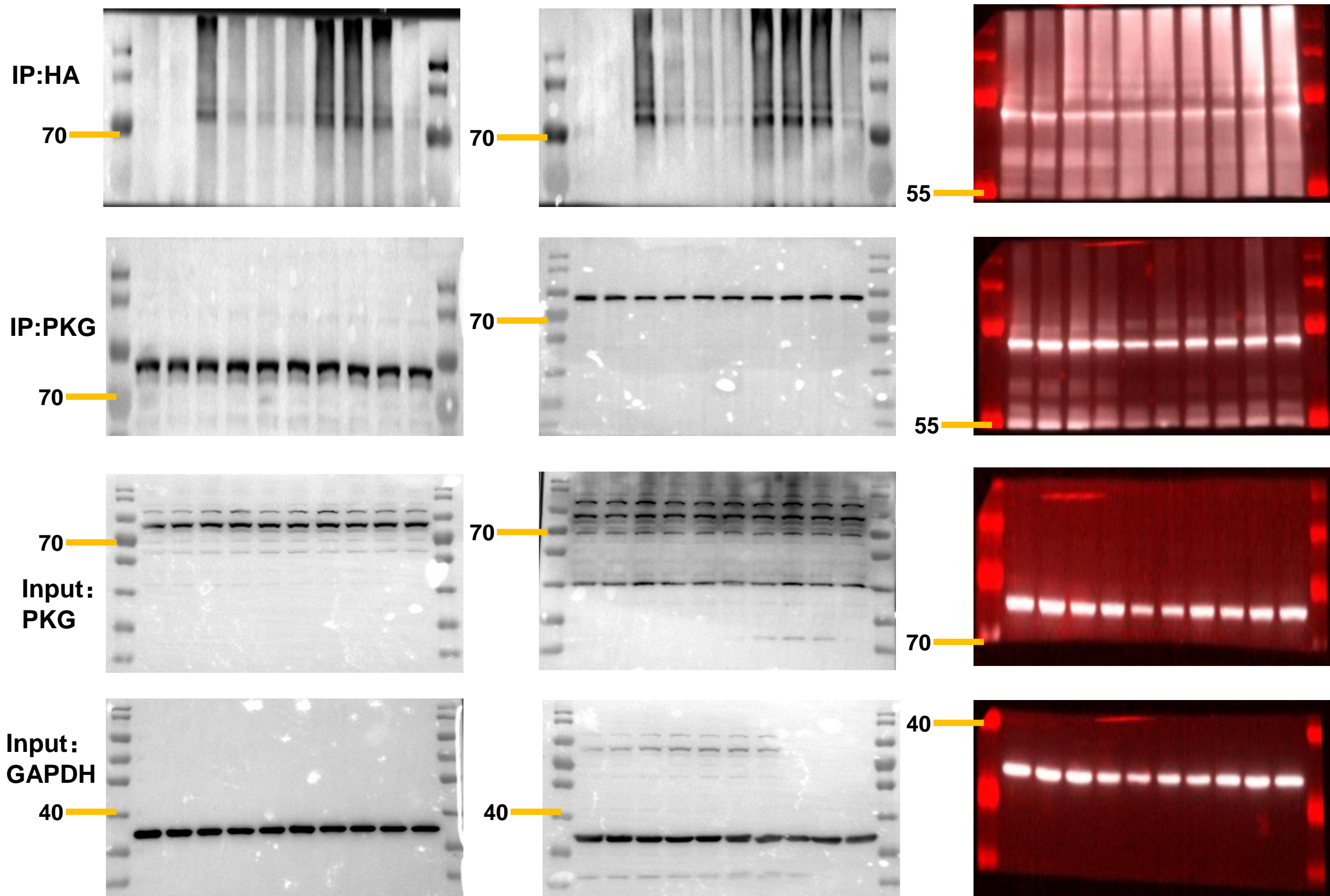

Supplement: Supplementary file 16 — Supplementary file16 (PDF 526 KB) [file 395_2021_878_MOESM16_ESM.pdf]
