# Supplementary material for: Exercise-induced peptide TAG-23 protects cardiomyocytes from reperfusion injury through regulating PKG–cCbl interaction
Source: Basic Res Cardiol. 2021 Jun 25;116(1):41. doi: 10.1007/s00395-021-00878-4 (PMC8233271; doi:10.1007/s00395-021-00878-4)

6A H9C2

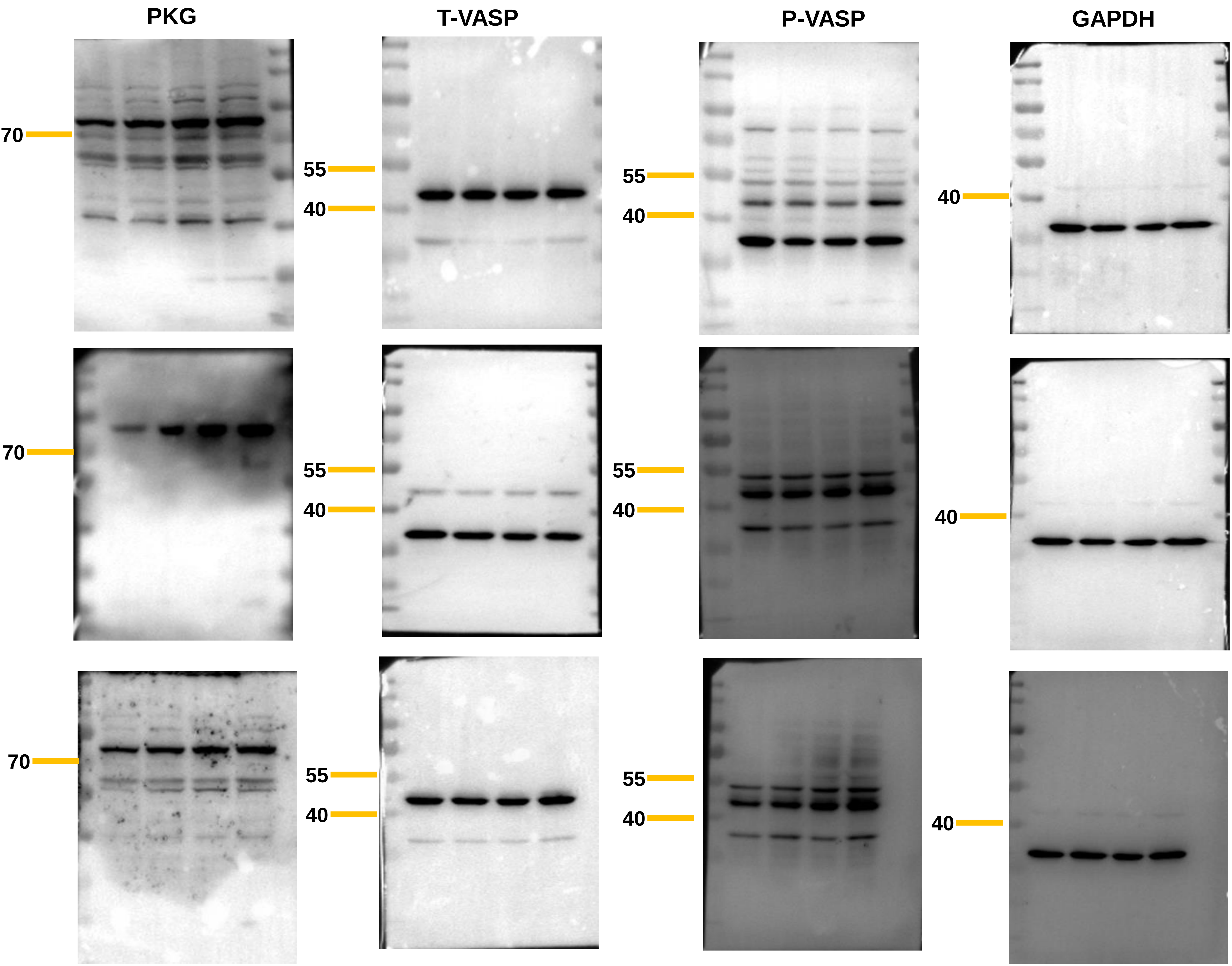

6B H9C2

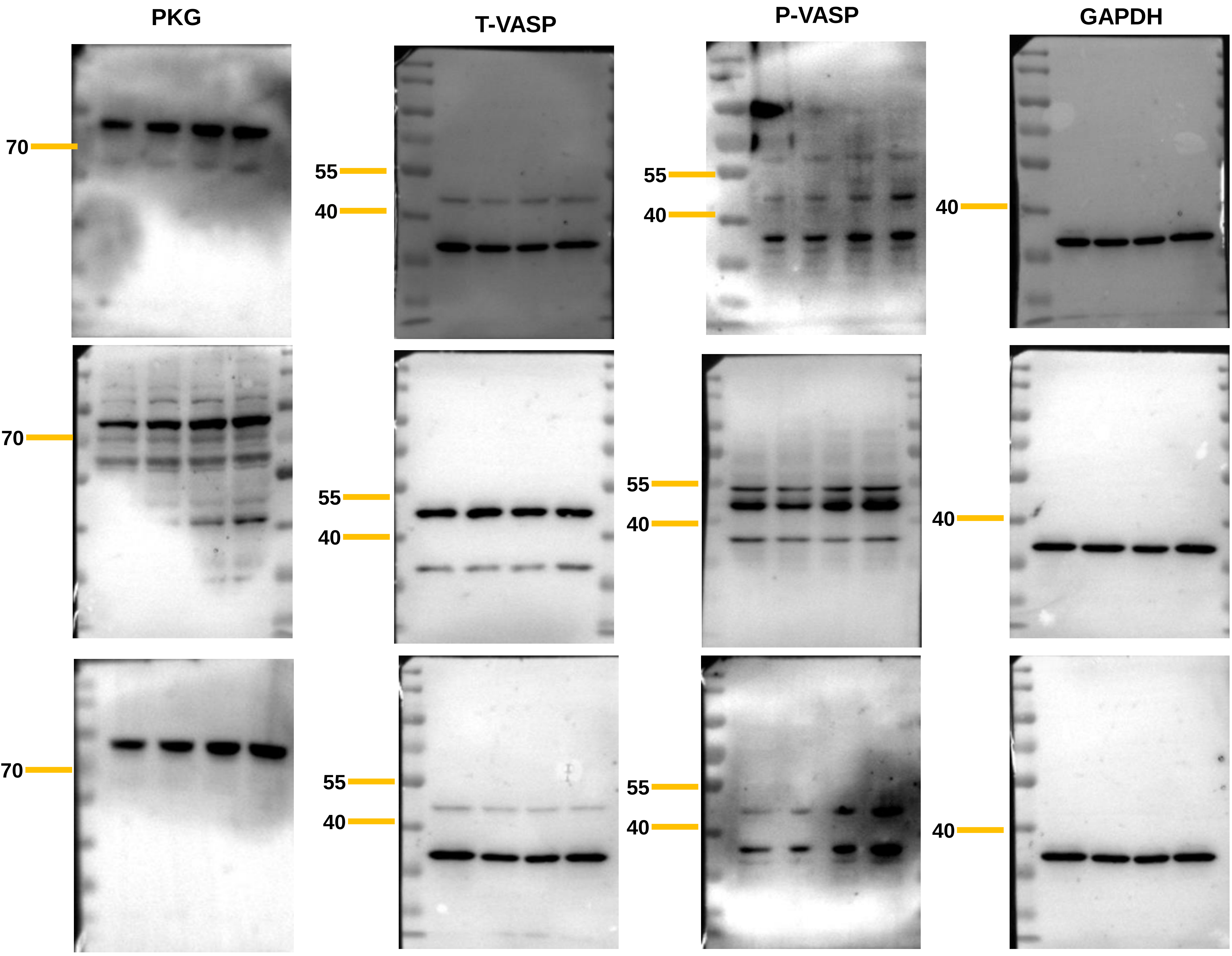
PKG
T-VASP
P-VASP
GAPDH
70
55
40
55
40
40
70
55
40
55
40
40
70
55
40
55
40
40

6C HEK293

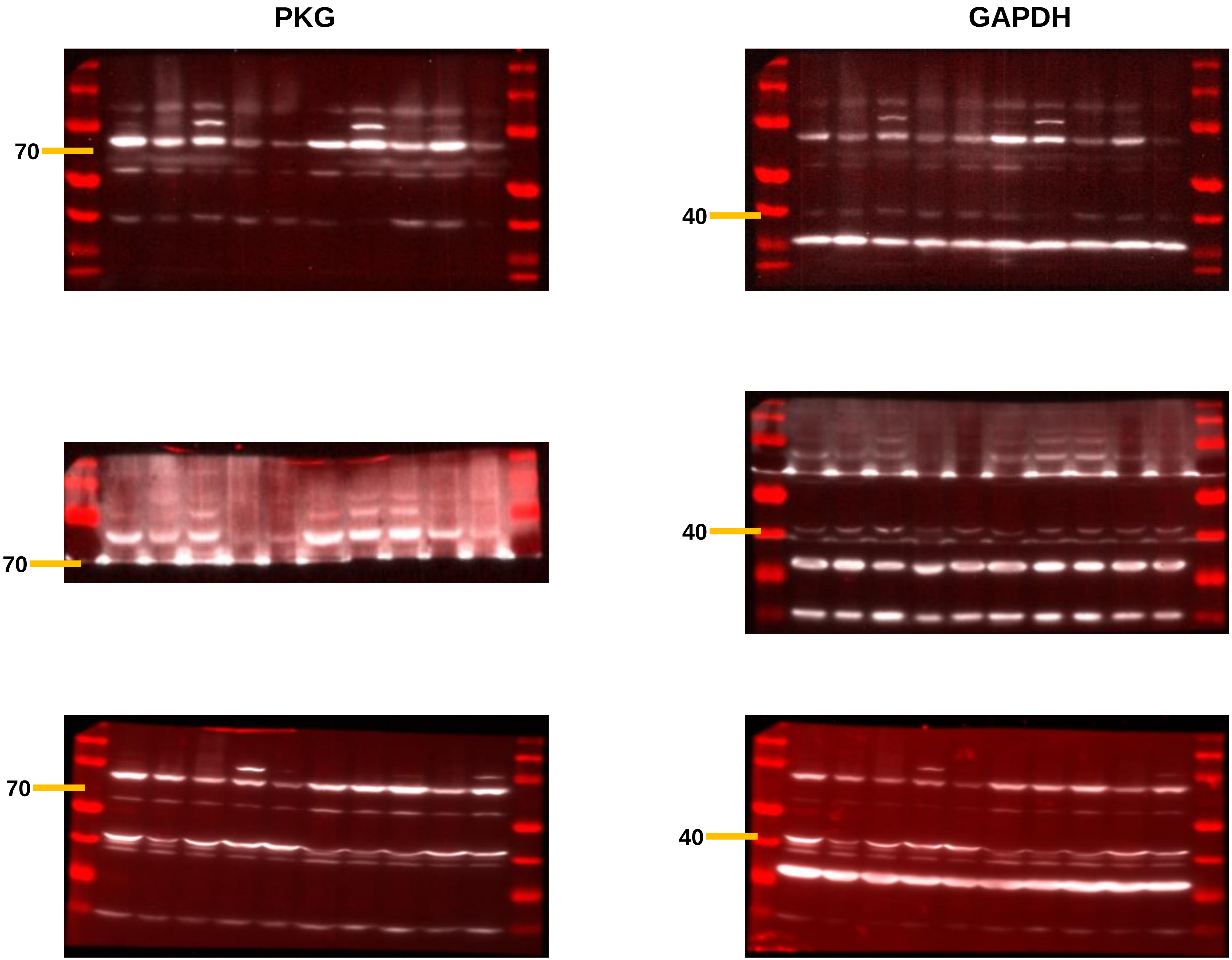

6C H9C2

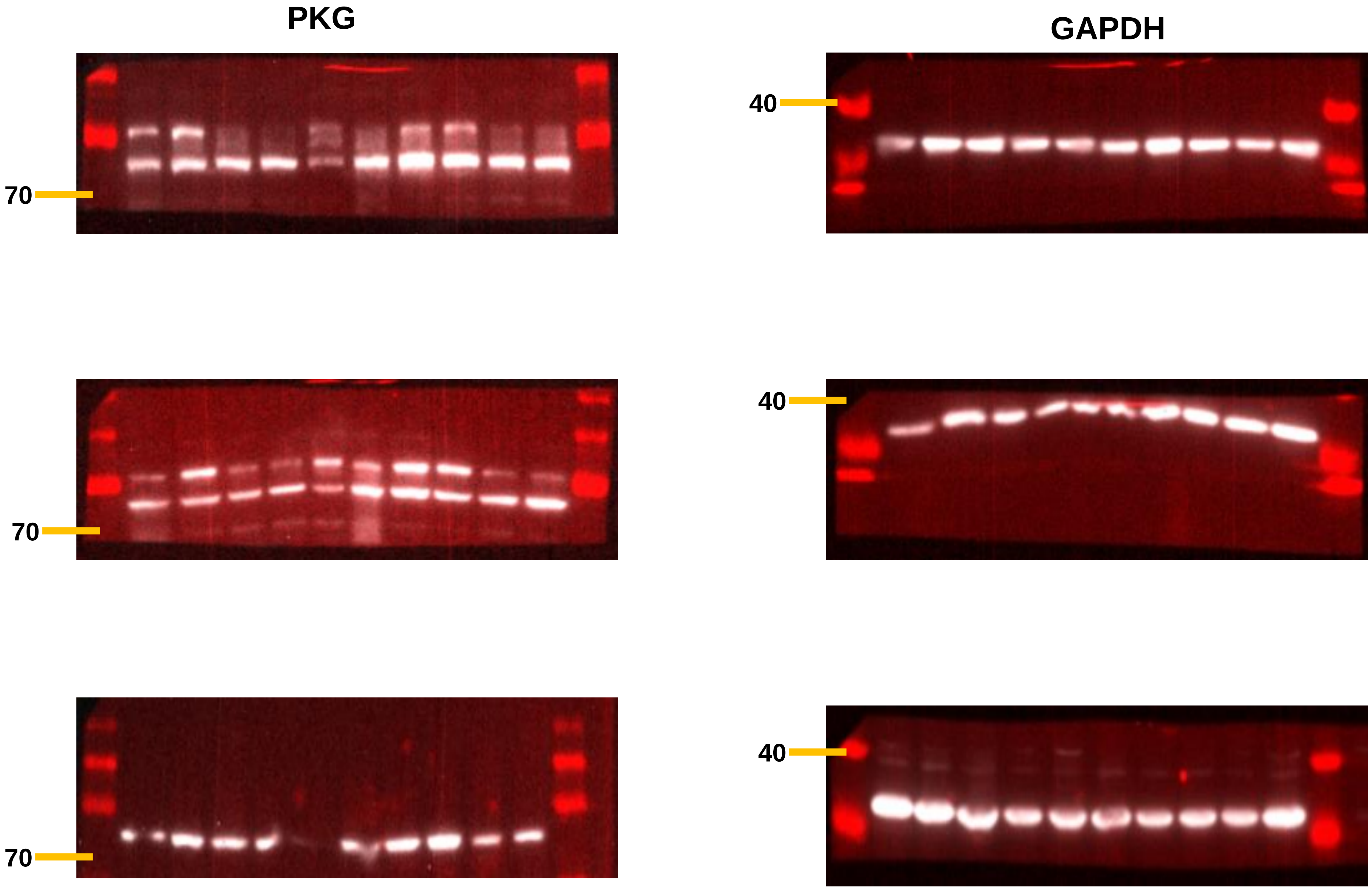

6D HEK293

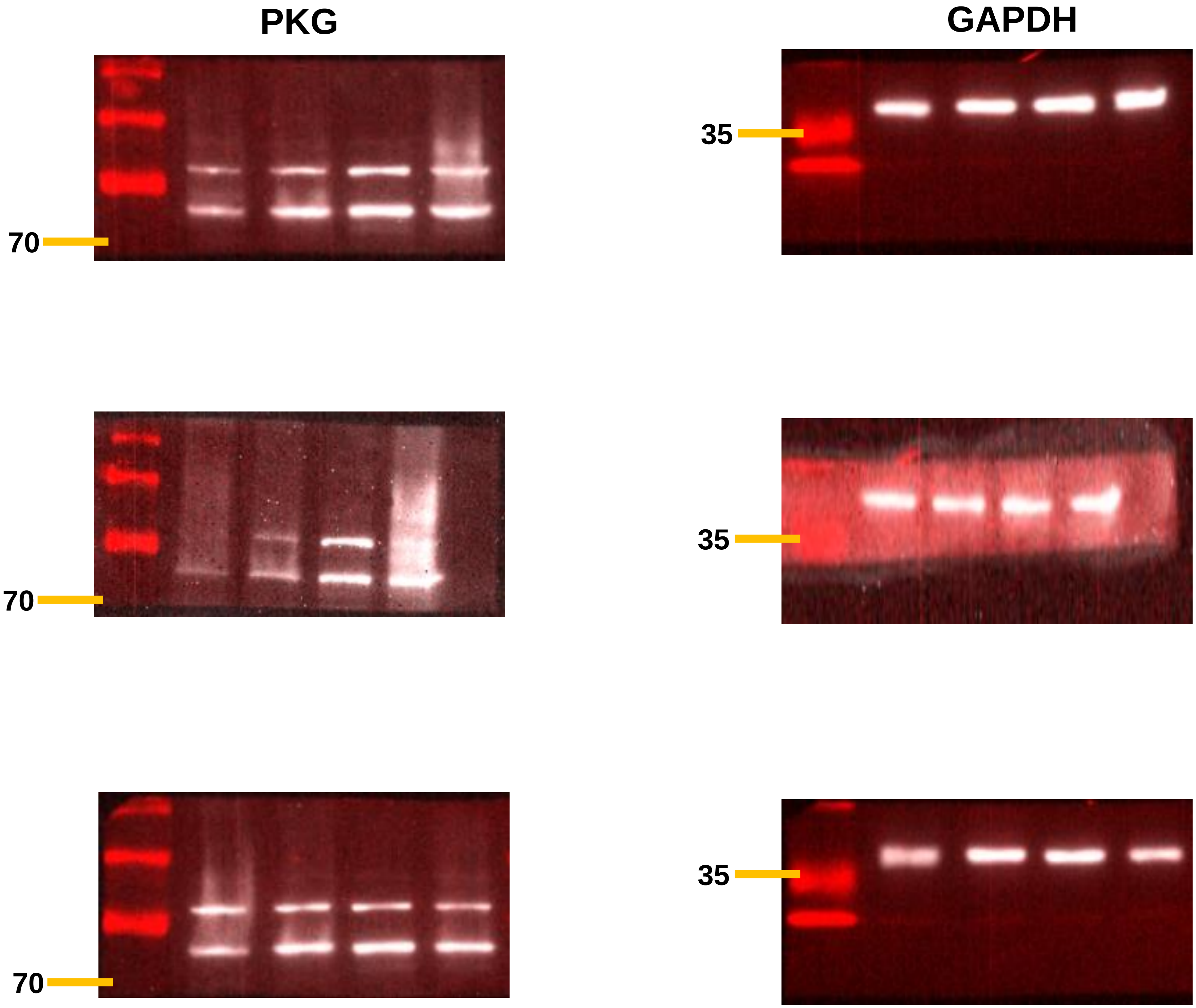

6D H9C2

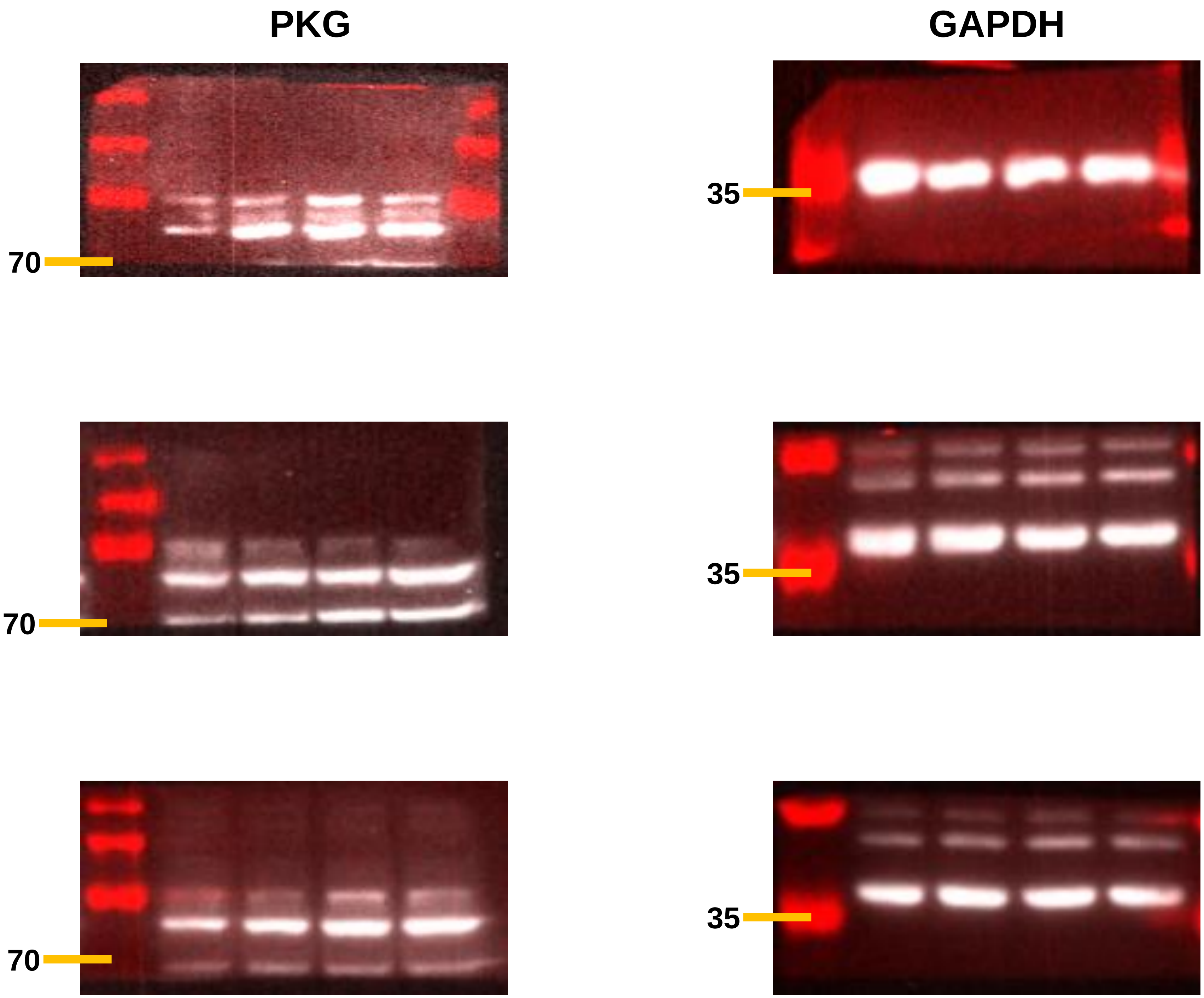

6E H9C2

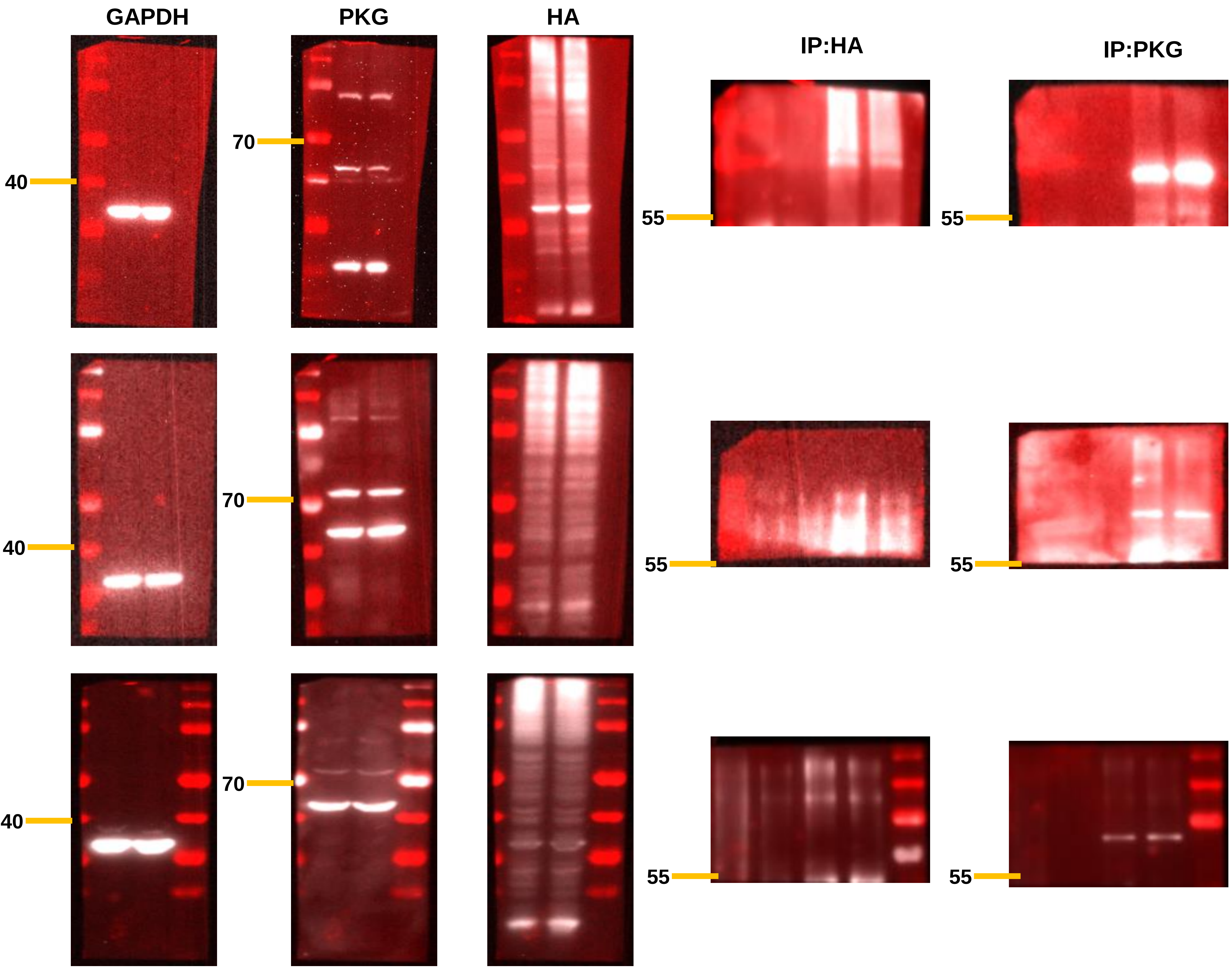

6F HEK293

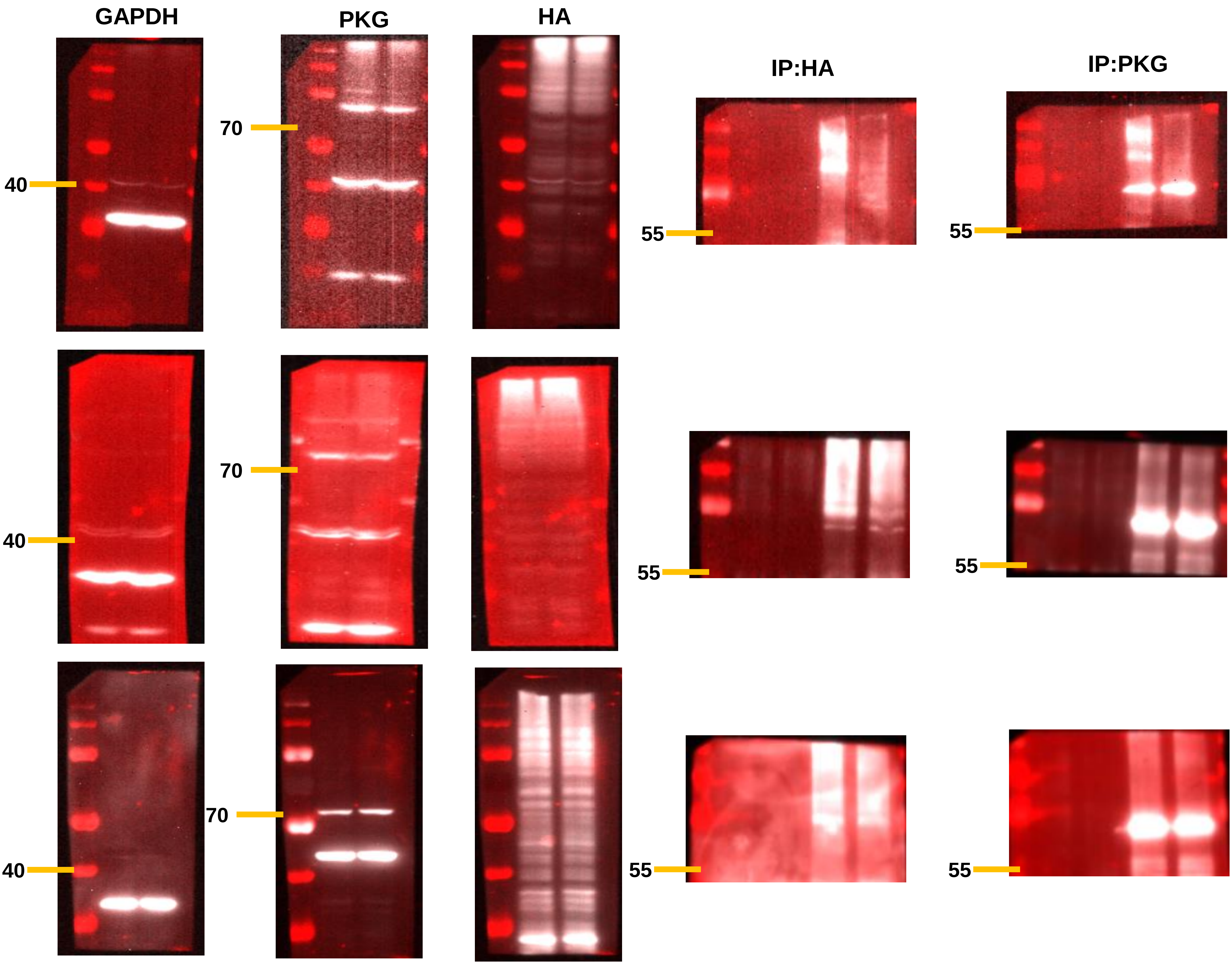

Supplement: Supplementary file 14 — Supplementary file14 (PDF 570 KB) [file 395_2021_878_MOESM14_ESM.pdf]
